# SARS-CoV-2 infects neurons and induces neuroinflammation in a non-human primate model of COVID-19

**DOI:** 10.1016/j.celrep.2022.111573

**Published:** 2022-10-12

**Authors:** Danielle Beckman, Alyssa Bonillas, Giovanne B. Diniz, Sean Ott, Jamin W. Roh, Sonny R. Elizaldi, Brian A. Schmidt, Rebecca L. Sammak, Koen K.A. Van Rompay, Smita S. Iyer, John H. Morrison

**Affiliations:** 1 California National Primate Research Center, University of California Davis, Davis, CA 95616, USA; 2 Center for Immunology and Infectious Diseases, University of California Davis, Davis, CA 95616, USA; 3 Graduate Group in Immunology, University of California Davis, Davis, CA 95616, USA; 4 Department of Pathology, Microbiology, and Immunology, School of Veterinary Medicine, University of California Davis, Davis, CA 95616, USA; 5 Department of Neurology, School of Medicine, University of California Davis, Davis, CA 95616, USA

**Keywords:** coronavirus, neurotropism, microglia, astrocytes, rhesus, NHP, macaque

## Abstract

Severe acute respiratory syndrome coronavirus 2 (SARS-CoV-2), the etiologic agent of coronavirus disease 2019 (COVID-19), can induce a plethora of neurological complications in some patients. However, it is still under debate whether SARS-CoV-2 directly infects the brain or whether CNS sequelae result from systemic inflammatory responses triggered in the periphery. By using high-resolution microscopy, we investigated whether SARS-CoV-2 reaches the brain and how viral neurotropism can be modulated by aging in a non-human primate model of COVID-19. Seven days after infection, SARS-CoV-2 was detected in the olfactory cortex and interconnected regions and was accompanied by robust neuroinflammation and neuronal damage exacerbated in aged, diabetic animals. Our study provides an initial framework for identifying the molecular and cellular mechanisms underlying SARS-CoV-2 neurological complications, which will be essential to reducing both the short- and long-term burden of COVID-19.

## Highlights

• In macaques, SARS-CoV-2 is found in olfactory brain areas at 7 days post infection
• Neurons are initially the primary target of SARS-CoV-2 productive infection
• Neurocovid is accompanied by robust neuroinflammation and vascular disruption
• SARS-CoV-2 brain pathology is worsened by aging and diabetes in infected monkeys

## Graphical abstract

**Figure:**
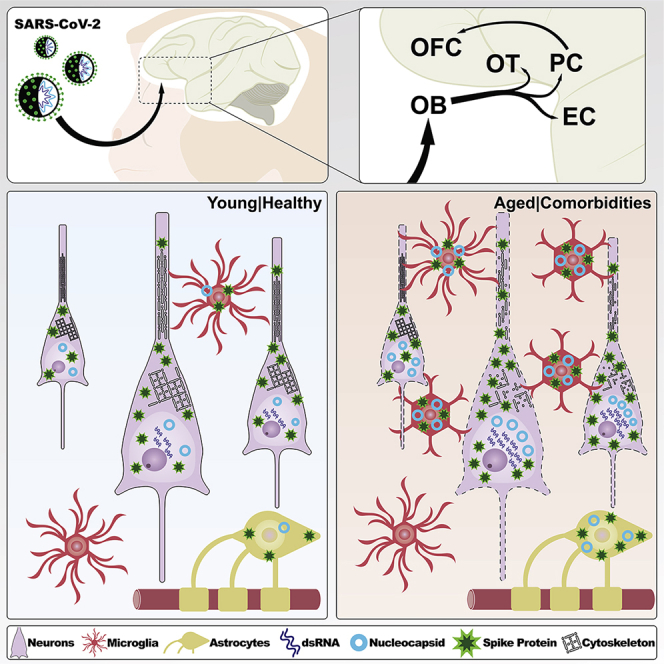

## Teaser

Beckman et al. show that SARS-CoV-2 proteins are found in olfactory areas of the brains of rhesus macaques at 7 days post infection. In addition to direct neuronal infection, extensive neuroinflammation and vascular disruptions are observed, and these effects are exacerbated in aged, diabetic animals.

## Introduction

Coronaviruses, members of the subfamily *Orthocoronaviridae*, are enveloped single-stranded RNA viruses known to infect a wide range of bird and mammalian species, including humans. In 2019, the newly emerged coronavirus severe acute respiratory syndrome coronavirus 2 (SARS-CoV-2) became a significant public health concern, infecting over 96 million people in the United States alone and resulting in more than one million deaths, disproportionally affecting older individuals with preexisting comorbidities.^1^ In addition to respiratory and gastrointestinal symptoms commonly caused by other coronaviruses, SARS-CoV-2 infection is accompanied by a myriad of neurological presentations in up to 80% of hospitalized patients, in addition to reductions in gray-matter thickness and tissue contrast observed through *in vivo* imaging.^2^^,^^3^ Considering the high degree of homology between SARS-CoV-2 and other coronaviruses with demonstrated neurotropic potential, including SARS-CoV-1 and Middle Eastern respiratory syndrome coronavirus (MERS-CoV),^4^^,^^5^^,^^6^ there is a critical need to determine if a direct infection of the central nervous system by SARS-CoV-2 is the underlying mechanism for the neurological symptoms in coronavirus disease 2019 (COVID-19).

Direct examination of post-mortem brain samples from patients with COVID-19 has yielded contradictory results, with several studies reporting the positive detection of viral RNA in the CNS,^7^^,^^8^ although others found low or undetectable viral RNA levels^9^^,^^10^^,^^11^ These variations underscore the need for animal models that may allow us to probe SARS-CoV-2 behavior in a more controlled environment, with rhesus macaques (*Macaca mulatta*) showing great potential as a platform for scientific discovery in COVID-19. These animals have extensive similarities with human immunological responses and have already significantly contributed to defining the safety and effectiveness of SARS-CoV-2 vaccines, uniquely positioning them to help us understand the effects of COVID-19 in the CNS.^9^^,^^12^

Considering the above, the California National Primate Research Center (CNPRC) has launched an ongoing effort to comprehensively characterize SARS-CoV-2 infection in macaques, including its neurotropic potential.^13^^,^^14^^,^^15^ In this study, we aimed to establish whether SARS-CoV-2 can be detected in the brains of rhesus monkeys during the acute phase of the infection by employing high-resolution three-dimensional (3D) confocal microscopy combined with extensive morphometric analyses using semi-automated object segmentation.^16^^,^^17^ Toward that goal, we intranasally and intratracheally inoculated young, healthy rhesus monkeys (4–6 years old; n = 4) with a high dose of SARS-CoV-2 (2.5 × 10^6^ plaque-forming units [PFUs]) and euthanized the animals at 7 days post infection (7 dpi)^14^ (Figure S1). Additionally, to better understand how age and comorbidities may contribute to CNS infection, we included an additional cohort of type II diabetic (T2D), aged monkeys (18–24 years old; n = 4) subject to the same experimental design. Non-infected, aged-matched animals without T2D (young n = 2; aged n = 2) and with T2D (young n = 1; aged n = 1) were included in the study as biological controls. Chronic illness and current treatments, as well as COVID-19 pathology developed by infected animals, are presented in Table S1 and Data S1. Notably, at the time of the brain analyses, COVID-19 clinical signs of infection were generally mild and did not require intervention (more details in Shaan Lakshmanappa et al.^14^ and Rompay et al.^13^). A summary of the antibodies used in this study can be found in Figure S1.

## Results

### SARS-CoV-2 markers are found in olfactory areas in an age-dependent manner at 7 dpi

**Figure 1:**
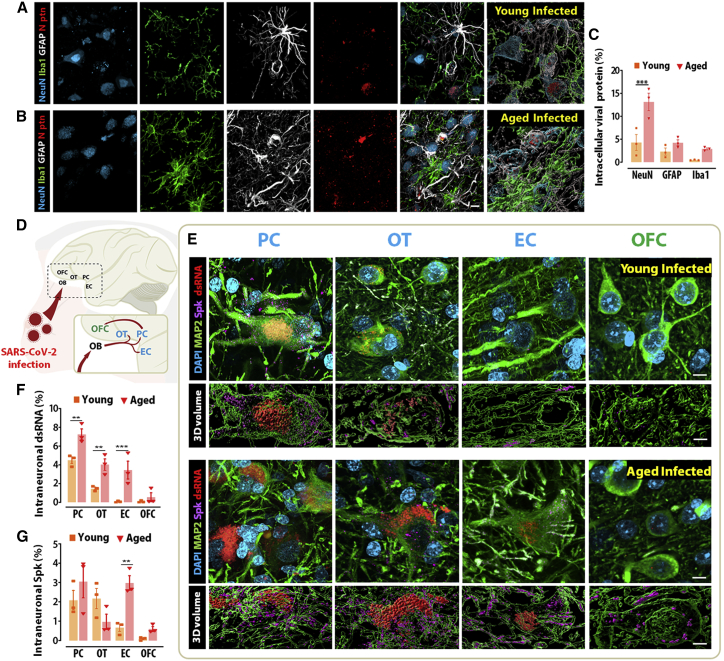
SARS-CoV2 preferentially infects neurons and spreads faster throughout the olfactory cortex of aged, infected monkeys (A and B) Quantitative quadruple staining combining markers for neuron (NeuN), microglia (Iba1), and astrocytes (GFAP) with SARS-CoV2 nucleocapsid protein (N ptn) was performed in the primary olfactory cortex of young and aged infected monkeys. (C) Internalized N ptn volume was calculated in 3D and divided by the total 3D volume obtained for each infected cell type analyzed. (D) The neurotropic potential of SARS-CoV2 was investigated in the primary olfactory cortex (blue; piriform cortex [PC], olfactory tubercle [OT], and entorhinal cortex [EC]) and the prefrontal secondary olfactory region, the orbitofrontal cortex (OFC; green). (E) Representative micrographs and 3D volume reconstruction show spike (Spk) protein (purple) and dsRNA (red) expression across several olfactory regions. (F and G) Quantification of the intraneuronal levels of dsRNA (F) and Spk protein (G) demonstrates that SARS-CoV-2 spreads faster in aged animals compared with young, infected controls. Scale bar, 50 μm. ^∗∗^p < 0.01, ^∗∗∗^p < 0.001, two-way ANOVA, Sidak’s post hoc test. Numerical data are represented as mean ± SEM. See also Figures S2 and S3 and Video S1.

Initial immunohistochemical labeling of SARS-CoV-2 nucleocapsid (N) protein in the frontal lobe revealed substantial immunoreactivity to SARS-CoV-2 N, demonstrating the presence of viral proteins in the brain within 7 days of inoculation. For the purposes of this section, results are reported for the piriform cortex, as this cortical area showed the highest immunohistochemical signal in preliminary results. Cell-type analysis revealed that the N immunosignal colocalized with all three major cell types within the brain, neurons (NeuN+), astrocytes (GFAP+), and microglia (Iba1+), in both young and aged animals (Figure 1A and 1B). Volume quantification of intracellular N protein by cell type indicates that neurons showed the highest degree of intracellular N regardless of age, and aged animals displayed higher volumes compared with young animals across all three cell types, neurons (4.3% versus 13%; p = 0.0004), astrocytes (2.3% versus 4.2%; p = 0.5836), and microglia (0.5% versus 2.9%; p = 0.4034), although only neurons reached statistical significance (Figures 1C and S2). These results suggest a partial neurotropism for SARS-CoV-2 and an age-dependent increase in infection burden.

Following the preliminary establishment of SARS-CoV-2 entry into the CNS, we expanded the analysis to include different viral markers and additional brain areas. In addition to SARS-CoV-2 N, we were able to detect SARS-CoV-2 spike (Spk) protein and double-stranded RNA (dsRNA), an intermediary molecule in the replication cycle of SARS-CoV-2 that serves as a proxy for productive infection.^18^ Immunolabeling for these markers was found in multiple areas of the primary olfactory cortex, including the olfactory tubercle, the piriform cortex, and the olfactory pole of the entorhinal cortex, irrespective of age. In aged animals, SARS-CoV-2 viral markers were also observed in the orbitofrontal cortex (area 14), which is part of the secondary olfactory cortex. In contrast, only minimal labeling was observed outside the primary olfactory cortex in young animals (Figures 1D, 1E, and S3). This distribution pattern is coherent with the axonal spread of the virus from the nasal olfactory epithelium, a mechanism of entry previously described for other coronaviruses.^19^^,^^20^^,^^21^

Since the distribution pattern of SARS-CoV-2 Spk and, in particular, dsRNA was predominantly neuronal (Video S1), we proceeded to perform a regional morphometric analysis of these markers in neurons of the abovementioned areas. Across all sites, dsRNA immunoreactivity was detected exclusively within the soma, while Spk immunoreactivity was found predominantly within dendrites (Figure S2). Compared with aged control (CTR) animals, infection with SARS-CoV-2 in older animals was associated with a significant decrease in cell body volume (p = 0.0063) and a trend for reduced dendrite volume (p = 0.0571) (Figure S2). Across the primary olfactory cortex, the percentage of intraneuronal dsRNA was significantly higher in aged animals compared with young animals: piriform cortex (4.4% versus 7.1%, p = 0.0049), olfactory tubercle (1.4% versus 3.9%, p = 0.0091), and entorhinal cortex (0.08% versus 3.4%, p = 0.0008). No statistical difference was observed in the orbitofrontal cortex (0.1% versus 0.5%, p = 0.9453), likely due to the very low overall signal observed in both young and aged animals (Figure 1F). While a similar pattern of higher intraneuronal localization was also observed for Spk, higher intragroup variability led to a statistically significant difference between young and aged animals exclusively in the entorhinal cortex (0.7% versus 3%; p = 0.0081) (Figure 1G).

**Figure 2:**
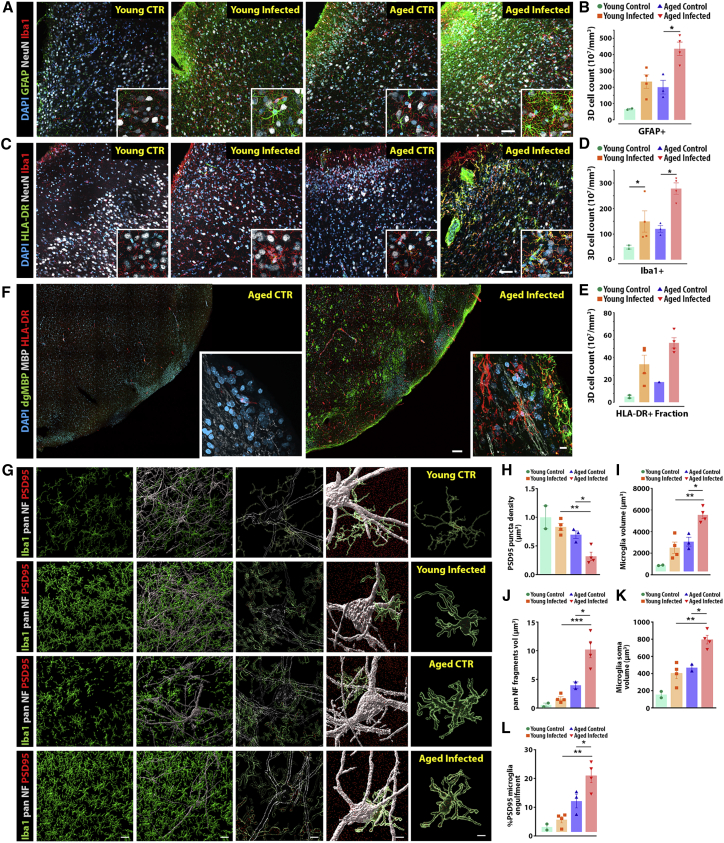
The highly connected olfactory cortex shows a robust inflammatory response following SARS-CoV-2 infection (A and B) Unbiased 3D microscopic quantification shows a significant increase in astrocyte recruitment (GFAP+) following SARS-CoV-2 infection. (C and E) In addition, a robust increase in general (Iba1) and activated (HLA-DR) microglia markers are also associated with aging. (F) HLA-DR+ reactive microglia were also found associated with degraded myelin basic protein (dgMBP), resulting in reduced normal myelin basic protein (MBP) expression in aged-infected animals in comparison with aged controls. (G) To further analyze neuron-microglia dynamics across the experimental groups, we combined general markers for neurons (pan-NF) and microglia (Iba1) with the post-synaptic marker PSD95 in the primary olfactory region. (H–L) Analysis of PSD95 puncta density (H), microglial total cell volume (I), pan-NF microglial engulfment (J), microglia soma volume (K), and PSD95 microglial engulfment (L) demonstrates a robust phagocytic response observed in the brain following SARS-CoV-2 infection, especially in the older infected population. Scale bars, 200 μm (A–F), 10 μm, and 5 μm (3D) (G). ^∗^p < 0.05, ^∗∗^p < 0.01, ^∗∗∗^p < 0.001, one-way ANOVA, Tukey’s post hoc test. Numerical data are represented as mean ± SEM. See also Figures S4–S7 and Video S1.

Video S1. Three-dimensional representation of key findings of the study, related to Figures 1, 2, and 3 Video 01 (00:00) – 3D representation of SARS-CoV-2 viral proteins (dsRNA – red, SARS-CoV-2-Spk – purple) within a neuron (MAP2 – green, DAPI - blue) located in layer II of the entorhinal cortex of an aged, infected animal. Double-stranded RNA is found within the soma, closely associated with its degrading nucleus, while SARS-CoV-2-Spk is found distributed throughout the surface of the soma and of MAP2+ processes; Video 02 (00:45) - Three-dimensional representation of SARS-CoV-2 viral proteins (dsRNA – red, SARS-CoV-2-Spk – purple) within a neuron (MAP2 – green, DAPI - blue) located in layer II of the entorhinal cortex of an aged, infected animal. The presence of SARS-CoV-2 within a neuron is associated with significant suppression of MAP2 immunosignal, suggesting infection is associated with decreased neuronal integrity. Video 03 (01:30) - Three-dimensional representation of SARS-CoV-2 viral proteins (dsRNA – red, SARS-CoV-2-Spk – purple) within neurons (MAP2 – green, DAPI - blue) located in the piriform cortex of an aged, infected animal. Different degrees of dsRNA immunosignal can be found in neurons within the piriform cortex, suggesting a progressive replication process following SARS-CoV-2 infection accompanied by neuronal degeneration; Video 04 (02:10) - Three-dimensional representation of astrocytes (GFAP – white) and microglia (IBA1 – green) contacting neurons (NeuN – blue) positive for nucleocapsid protein (SARS-CoV-2-N – red) in the olfactory cortex of a young, infected animal. The directed contact between infected neurons and glial cells suggests that infection triggers a neuroinflammatory response that may contribute to neuronal death; Video 05 (02:55) - Three-dimensional representation of microglia (IBA1 – red, DAPI - blue) interacting with neurons (Pan Neurofilament – white, DAPI – blue) and dendritic spines (PSD95 – green) in the primary olfactory cortex of an infected animal. The direct contact between microglia and neurons in infected areas suggests a direct action of microglia over neuronal degeneration, a mechanism underscored by the substantial engulfment of PSD95 + puncta; Video 06 (03:35) - Three-dimensional representation of microglia (IBA1 – red) interacting with neurons (Pan Neurofilament – white) and dendritic spines (PSD95 – green) in the piriform cortex of an infected animal, indicating extensive dendritic spine engulfment by activated microglia in SARS-CoV-2-bearing areas; Video 07 (04:10) - Three-dimensional representation of an activated neutrophil (Myeloperoxidase – red, DAPI - blue) recruitment associated with blood-brain barrier disruption, as evidenced by disorganization of vascular astrocytic endfeet (GFAP – green), among neurons (NeuN – white, DAPI – blue) of the primary olfactory cortex of a SARS-CoV-2 infected animal; Video 08 (04:47) - Three-dimensional representation of an activated neutrophil (Myeloperoxidase – white, DAPI - blue) recruitment associated with blood-brain barrier disruption, as evidenced by disorganization of vascular astrocytic endfeet (GFAP – red), among neurons (NeuN – green, DAPI – blue) of the primary olfactory cortex of a SARS-CoV-2 infected animal.

Taken together, our data indicate that SARS-CoV-2 is neurotropic and can be detected in olfactory areas of the macaque brain within 7 dpi. Viral proteins are found in both neuronal and glial cells, but productive infection at this time point is predominantly neuronal. Aged T2D animals displayed increased viral load across all areas examined, in addition to more pronounced cellular alterations in response to the virus.

### SARS-CoV-2 infection leads to neuroinflammation that is exacerbated in aged animals

Although neurotropic viruses have developed mechanisms to escape host immune surveillance and facilitate CNS entry,^22^ local inflammation is still a factor contributing further to neuronal damage and death and may offer an addressable therapeutic target. Therefore, we investigated neuroinflammation in our rhesus model of COVID-19. Informed by the results described above, we focused our analysis on the piriform cortex, which consistently displayed the highest SARS-CoV-2 Spk and dsRNA levels.

We detected an increase in the number of astrocytes between young, infected animals and their age-matched, non-infected controls (p = 0.0581). Notably, a significant increase in the GFAP population was observed comparing young and aged infected animals (p = 0.011), suggesting either proliferation or translocation of astrocytes to the piriform cortex. In addition, a robust increase in astrocytic 3D cell volume was also observed when comparing young and aged infected animals (p = 0.0009) (Figures 2A, 2B, S2, and S4). A similar pattern of activation was detected for microglia, as measured by the fraction of Iba1+ cells coexpressing the major histocompatibility complex (MHC) class II surface receptor HLA-DR (p = 0.0344) (Figures 2C–2E, S4, and S5). In addition, microglial proliferation/translocation in response to infection was significantly increased in aged, infected animals when compared with the young, infected group (p = 0.0049) (Figure 2D). These results suggest a robust inflammatory process in response to SARS-CoV-2 infection that mobilizes both astrocytic and microglial cascades (Figure S4). Notably, investigation of the piriform cortex of diabetic young and aged control animals detected inflammatory alterations associated with diabetes only in the aged animals (Figures S4 and S5).

With normal aging, microglia acquire a hypersensitive state that can quickly shift to an inflammatory profile upon viral infection, increasing the risk of developing neurodegenerative diseases.^23^^,^^24^ Several lines of evidence indicate a similar shift takes place in the animal model described in this work: (1) HLA-DR+ microglia were found associated with degraded myelin basic protein (dgMBP) at the expense of regular MBP expression, potentially leading to white-matter injury (Figures 2F and S6); (2) HLA-DR+ microglia were found in contact with neurons expressing MHC class I, a viral antigen substantially enhanced in neurons from diabetic older animals, but worsened following SARS-CoV-2 infection (Figure S5); and (3) there is a higher frequency of microglia with abnormal morphology (e.g., truncated processes, round soma) in infected animals, and these microglia are often found adjacent to neurons with morphological changes, suggesting profound impact suggestive of neurodegenerative processes (Figures 2G, S7, and S8; Video S1).

Considering these findings, we hypothesized that SARS-CoV-2 neuroinflammation might lead to synaptic and cellular damage. Consistent with that idea, we observed a significant decrease in the density of PSD95+ puncta in infected, aged animals compared with young, infected ones (p = 0.0081) (Figure 2H). The total microglia cellular volume from aged, infected animals was also increased when compared with microglia from young, infected ones (p = 0.0015), as well as when compared with aged, non-infected controls (p = 0.0023) (Figure 2I). Notably, aged, infected microglia also presented an increase in the total volume of internalized pan-Neurofilament (pan-NF) fragments (young, infected versus aged, infected: p = 0.0003, aged, CTR versus aged, infected: p = 0.0019) (Figure 2J). Increased microglial soma volume (young infected versus aged infected: p = 0.0011, aged, CTR versus aged, infected: p = 0.0029) (Figure 2K) was also detected and correlated with selective microglia-engulfed PSD95 (young, infected versus aged, infected: p = 0.0005, aged, CTR versus aged, infected: p = 0.0337) (Figure 2L), suggesting a direct effect of activated microglia over the reduction in synaptic boutons (Figure 2I). A summary of the 3D analysis protocol used in this study can be found in Figure S8.

In summary, SARS-CoV-2 infection leads to a fast cascade of inflammatory events driven by astrocytic and microglial responses. In both cases, aged animals showed comparatively higher levels of inflammation than infected young animals. In addition, a significant increase in microglia density and synaptic engulfment were observed exclusively in older, infected animals, suggesting a compounding effect of aging and antiviral response. Notably, synaptic pruning and myelin degradation mediated by microglia may underlie some of the neurological deficits observed in patients with COVID-19.

### SARS-CoV-2 interacts with ACE2 and leads to local disruption of vascular homeostasis

Similar to SARS-CoV-1, the SARS-CoV-2 Spk protein binds to the membrane-bound angiotensin-converting enzyme 2 (ACE2) with great affinity to initiate the cell entry process. Human ACE2 shares a unique profile identity with only three other species, one of them being the rhesus monkey,^25^ prompting us to explore ACE2-Spk binding in a highly translatable model of COVID-19 pathology.

**Figure 3:**
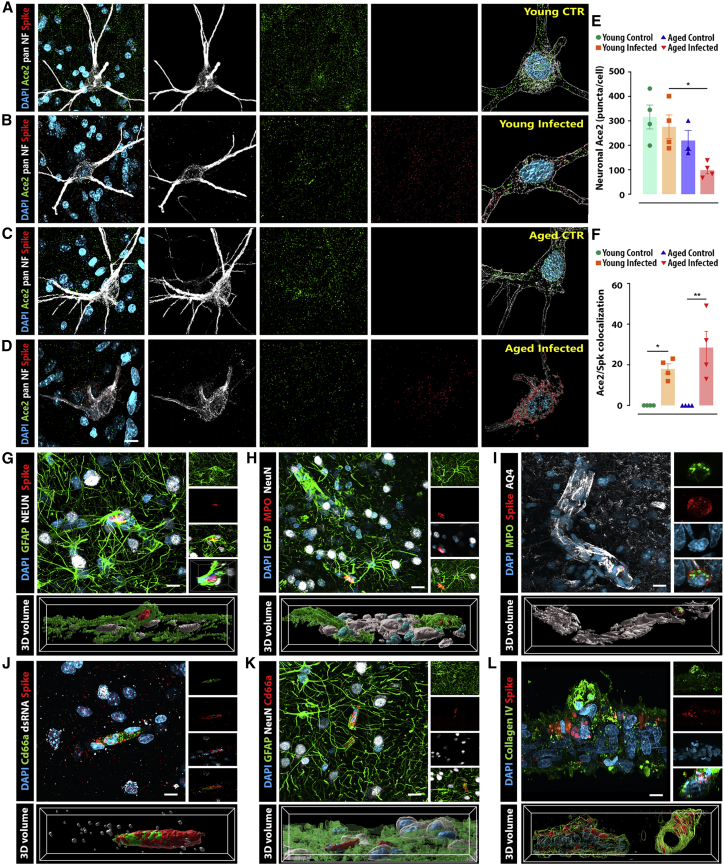
SARS-CoV-2 infection induces blood vessel disruption, reactive neutrophil recruitment, and ACE2 receptor downregulation (A–D) Airyscan super-resolution microscopy was applied to quantify ACE2 and Spk protein expression and binding within olfactory neurons (pan-NF maker) across the different experimental groups. (E) ACE2 expression is substantially reduced in the neurons of aged, infected animals compared with young, infected controls. (F) In addition, around 15%–40% of the remaining receptors colocalize with Spk protein in both infected groups. Multiple structural abnormalities in the BBB were observed across the olfactory cortex of infected monkeys. (G) Neurons (NeuN) and astrocytes (GFAP) present high levels of ACE2 expression and are also the major cell types expressing Spk protein. (H) Notably, disrupted blood vessels are associated with activated neutrophils (MPO) and robust recruitment of reactive astrocytes. (I) Altered BBB was also confirmed using aquaporin 4 (AQ4), a water channel expressed in astrocytic endfeet and involved in edema formation. (J and K) Additionally, the identity of neutrophils was confirmed using the CD66a marker, recruited to the injury. (L) Isolated blood vessels from surrounding meninges also show viral expression. Scale bars, 10 μm (A–D), 50 μm (G–K), and 5 μm (L). ^∗^p < 0.05, ^∗∗^p < 0.01, one-way ANOVA, Tukey’s post hoc test. Numerical data are represented as mean ± SEM. See also Figure S6 and Video S1.

As in the previous section, we have examined the neuronal expression of ACE2 in the piriform cortex due to the substantial viral presence in this cortical area compared with the other regions studied (Figures 3A–3D). Notably, a significant downregulation of ACE2 was observed in the aged, infected group when compared with young, infected animals (p = 0.0396). In addition, infected animals also presented, on average, 18% and 28% of ACE2 puncta colocalized with Spk immunosignal in young and aged animals, respectively (Figure 3F).

In addition to ACE2 downregulation, the presence of SARS-CoV-2 was also associated with morphological abnormalities in the astrocytic-vascular coupling necessary for the appropriate function of the blood-brain barrier (BBB). This apparent disruption in BBB integrity was often accompanied by reactive neutrophils (myeloperoxidase+) positive for Spk immunoreactivity (Figures 3G–3I and S6; Video S1). These results suggest that SARS-CoV-2 Spk may be associated with BBB disruption and the consequent entry of peripheral cells into the brain.

## Discussion

**Figure 4:**
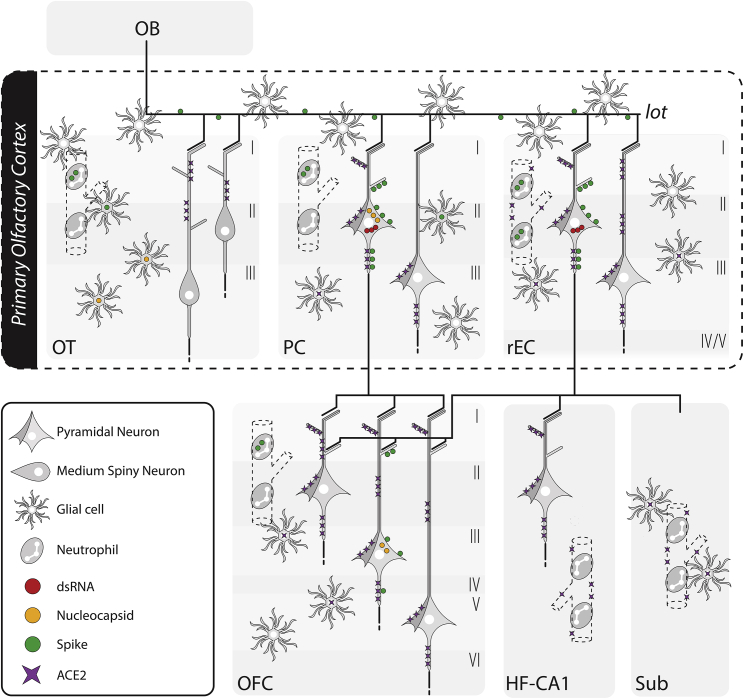
Summary of the anatomical localization, cell type, and viral protein spread observed in SARS-CoV-2-infected monkeys Schematic representation of the components of the olfactory circuit theorized to serve as the SARS-CoV-2-spreading pathway during the acute stage of the disease. The major viral proteins analyzed in this study were contained within the neural circuitry of the primary olfactory cortex, extending to the medial part of the OFC, a secondary olfactory region, only in aged monkeys. A robust immune response characterized by systemic neutrophil recruitment associated with disrupted blood vessels and local microglia and astrocyte activation was observed across all regions analyzed, including part of the hippocampal formation. I, cortical layer I; II, cortical layer II; III, cortical layer III; IV, cortical layer IV; V, cortical layer V; VI, cortical layer VI; ACE2, angiotensin-converting enzyme 2; dsRNA, double-stranded RNA; HF-CA1, hippocampal formation, CA1 field; mOFC, orbitofrontal cortex, medial part; OT, olfactory tubercle; PC, piriform cortex; rEC, entorhinal cortex, rostral subdivision; Sub, subicular complex.

In the present study, we have investigated the presence of SARS-CoV-2-related proteins in the brains of rhesus monkeys at 7 days post inoculation to see the neurotropic potential of SARS-CoV-2 at this early point in the course of the infection in a non-human primate. The overwhelming predominance of productive neuronal infection, combined with the spatially restricted distribution of viral proteins to the olfactory circuit, suggests the fast transneuronal spread of SARS-CoV-2 along corticocortical pathways, leading to its dissemination within the CNS via the olfactory connectome (summarized in Figure 4). This observation is in good agreement with previous reports that have demonstrated a similar progression pattern in laboratory animals for other human coronaviruses, such as HCoV-OC43 and HCoV-229E, and it closely matches the areas of decreased gray-matter thickness in human patients with COVID-19.^3^^,^^19^^,^^20^^,^^21^ Furthermore, the impact on olfactory pathways is consistent with the persistent anosmia observed in some patients with COVID-19.^26^ We cannot exclude, however, the possibility that the virus reaches the CNS through other pathways (e.g., vascular breakdown, translocation of infected immune cells) at later stages of the infection.

The results found in this work also indicate that the presence of SARS-CoV-2-related proteins within the olfactory pathway is accompanied by extensive microglial and astrocytic changes typically associated with neuroinflammatory responses, including alterations in cellular volume, morphology, and populational density, in addition to abnormal blood vessels and infiltration of activated neutrophils. Our observations are remarkably similar to those of Rutkai et al.,^27^ who found morphological changes in microglia consistent with activation, evidence of astrogliosis, and vascular homeostasis disruptions at 28 dpi. Combined, these works suggest that neuroinflammatory changes happen early during the course of the disease and can remain in place for an extended period of time, even if viral proteins are eliminated after the acute stage of the disease. Importantly, in aged animals, neuroinflammation was accompanied by synaptic engulfment and myelin degradation in areas exhibiting a high density of HLA-DR+ microglia, suggesting that demyelinating lesions and synaptic loss could be early deleterious effects resulting from an exacerbated neuroinflammatory response that can persist for several weeks. This process may explain why some patients with COVID-19 experience neurological symptoms, even in the absence of severe respiratory disease, and it underscores the potential for anti-neuroinflammatory agents in the control of COVID-19-related neurological sequelae.

The importance of the more widespread and severe neuronal infection by SARS-CoV-2 and the corresponding increased neuroinflammatory response observed in aged macaques with T2D and other comorbidities should not be understated. Older patients with diabetes (and other age-related comorbidities) are the most vulnerable population regarding COVID-19 severity and lethality, reinforcing the need to represent this complex group of individuals in translational animal models.^28^ Moreover, several early reports indicate that older patients experience neurological symptoms with increased severity and frequency, including complex neurological presentations unique to this population.^29^ Our work suggests that viral infection and senescence/comorbidities lead to synergistic damage to central nervous function, helping explain the uniqueness of this group regarding neurologic complications in COVID-19. We have also identified dsRNA and SARS-CoV-2 proteins in the entorhinal cortex of our experimental model of aged macaques, a cortical region particularly vulnerable to tauopathy associated with Alzheimer’s disease (AD). Considering the short period of time investigated in this work, it is likely that SARS-CoV-2 eventually reaches the same temporal and frontal cortical fields that are affected in AD, with yet unknown consequences. More studies will be necessary to understand the temporal course of the infection, as well as its implications for long-term neurological sequelae and potentially dementia.

### Limitations of this study

Some limitations of this study should be acknowledged. This study employs a group of aged animals (18–24 years old) with T2D combined with other comorbidities, an experimental group designed to mimic the clinical complexity of older human patients infected with SARS-CoV-2. Data obtained from these animals should be interpreted with care as there is an intrinsic challenge to dissociating the effects of aging, T2D, and other comorbidities. The limited supply of spontaneous T2D animals in our colony has constrained our ability to include more T2D controls, forcing us to compare data between aged, diabetic and aged, non-diabetic animals. Nevertheless, our diabetic controls have shown consistent results with those obtained in non-diabetic controls. The overall number of animals was also constrained as a whole due to cost and infrastructure limitations associated with keeping rhesus monkeys in A-BSL3 condition with complete veterinary care, even for a short period of time. To improve the chances of viral detection within the olfactory cortex 7 days after infection, we used a high dose of SARS-CoV-2 and inoculated the virus intranasally and intratracheally, another important caveat of this study. In addition, the olfactory epithelium and olfactory bulbs of these animals were unavailable at the time of this study, precluding us from drawing definitive conclusions about the route SARS-CoV-2 uses to access the brain, despite substantial evidence for an olfactory entry.

## STAR★Methods

### Key resources table

**Table:** 

REAGENT or RESOURCE	SOURCE	IDENTIFIER
**Antibodies**		

Rabbit polyclonal anti-amyloid oligomer	Sigma-Aldrich	Cat#AB9234; RRID: AB_11214948
Rabbit polyclonal anti-human ACE2	Abcam	Cat# ab15348; RRID: AB_301861
Goat polyclonal anti-ACE2	R&D Systems	Cat#AF933; RRID: AB_355722
Guinea pig polyclonal anti-Aquaporin 4	Synaptic Systems	Cat#429004; RRID: AB_2802156
Rat monoclonal anti-human CD66acd (clone YTH71.3)	Bio-Rad	Cat#MCA1147G; RRID: AB_2077339
Rat monoclonal anti-mouse CD8a (clone 53-6.7)	Invitrogen	Cat#14-0081-82; RRID: AB_467087
Rabbit monoclonal anti-Claudin-5 (clone EPR7583)	Abcam	Cat#ab131259; RRID: AB_11157940
Goat polyclonal anti-Collagen Type IV	Sigma-Aldrich	Cat#AB769; RRID: AB_92262
Rabbit polyclonal anti-Myelin basic protein, degraded	Us Biological	Cat#M9758-04; RRID: N/A
Mouse monoclonal anti-dsRNA (clone J2)	SciCons	Cat#10010200; RRID: AB_2651015
Chicken polyclonal anti-GFAP	Abcam	Cat#ab4674; RRID: AB_304558
Rabbit polyclonal anti-Iba1	Fujifilm Wako	Cat#019-19741; RRID: AB_839504
Mouse monoclonal anti-Iba1 (clone NCNP27)	Fujifilm Wako	Cat#013-27593; RRID: N/A
Guinea pig polyclonal anti-MAP2	Synaptic Systems	Cat#188004; RRID: AB_2138181
Rabbit monoclonal anti-human HLA-E (clone JF10-38)	Novus Bio	Cat#NBP2-66946; RRID: N/A
Mouse monoclonal anti-human HLA-DR (clone LN3)	BioLegend	Cat#327002; RRID: AB_893582
Mouse monoclonal anti-human HLA-DR (clone LN3)	Invitrogen	Cat#MA5-11966; RRID: AB_10979984
Human monoclonal anti-myelin basic protein (clone IGX3421)	Abcam	Cat#ab209328; RRID: AB_2818988
Mouse monoclonal anti-human myeloperoxidase (clone 2C7)	Bio-Rad	Cat#MCA1757; RRID: AB_2146467
Guinea pig polyclonal anti-NeuN	Synaptic Systems	Cat#266004; RRID: AB_2619988
Mouse monoclonal anti-NeuN (clone A60)	Sigma-Aldrich	Cat#MAB377; RRID: AB_2298772
Mouse monoclonal anti-Neurofilament H (clone SMI32)	BioLegend	Cat#801701; RRID: AB_2564642
Chicken polyclonal anti-Neurofilament heavy polypeptide	Abcam	Cat#ab4680; RRID: AB_304560
Mouse monoclonal anti-Neurofilament 70 kDa (clone DA2)	Sigma-Aldrich	Cat#MAB1615; RRID: AB_94285
Rabbit polyclonal anti-Olig-2	Sigma-Aldrich	Cat#AB9610; RRID: AB_570666
Mouse monoclonal anti-human Pan-Neuronal Neurofilament (clone TNJ-312)	Creative Diagnostics	Cat#DMAB7133; RRID: AB_2391764
Guinea pig polyclonal anti-Parvalbumin	Synaptic Systems	Cat#195004; RRID: AB_2156476
Goat polyclonal anti-PSD95	Abcam	Cat#ab12093; RRID: AB_298846
Mouse monoclonal anti-SARS/SARS-CoV-2 Nucleocapsid protein (clone E16C)	Invitrogen	Cat#MA1-7403; RRID: AB_1018420
Rabbit polyclonal anti-SARS-CoV-2 Spike Protein	Abcam	Cat#ab272504; RRID: AB_2847845
Human monoclonal anti-SARS-CoV-2 Spike Protein RBD (clone T01KHu)	Invitrogen	Cat#703958; RRID: AB_2866477
Guinea pig polyclonal anti-Synaptophysin 1	Synaptic Systems	Cat#101004; RRID: AB_1210382
Guinea pig polyclonal anti-Tight Junction Protein 1 (ZO-1)	Novus Bio	Cat#NBP1-49669; RRID: AB_10011789

**Bacterial and virus strains**		

SARS-CoV-2 2019-nCoV/USA-WA1/2020	BEI Resources	NR-52352

**Biological samples**		

Rhesus macaque CNS tissues (brain, vessels, leptomeninges, and choroid plexus)	CNPRC, University of California Davis	N/A

**Chemicals, peptides, and recombinant proteins**		

DAPI (4′,6-diamidino-2-phenylindole, dihydrochloride)	Invitrogen	Cat#D1306
ProLong Gold antifade reagent	Invitrogen	Cat#P36930
Bovine serum albumin	Sigma-Aldrich	Cat#A9647
Donkey serum	Sigma-Aldrich	Cat#D9663
Goat serum	MP Biomedicals	Cat#2939149
Target Retrieval Solution	Dako	Cat#S1700
Autofluorescence Eliminator Reagent	EMD Millipore	Cat#2160

**Experimental models: Organisms/strains**		

Rhesus macaque (Macaca mulatta)	CNPRC, University of California Davis	N/A

**Software and algorithms**		

ZEN (Blue Edition, version 2.3)	Carl Zeiss	https://www.zeiss.com/microscopy/us/products/microscope-software/zen.html
Imaris (version 9.8)	Bitplane/Oxford Instrumentals	https://imaris.oxinst.com/
Adobe Photoshop 2022	Adobe	https://www.adobe.com/products/photoshop.html
Adobe Illustrator 2022	Adobe	https://www.adobe.com/products/illustrator.html
GraphPad Prism (version 9)	GraphPad/Dotmatics	https://www.graphpad.com/scientific-software/prism/

### Resource availability

#### Lead contact

Additional information and requests for resources and reagents should be directed to and will be fulfilled by the Lead Contact, John H. Morrison (jhmorrison@ucdavis.edu).

#### Materials availability

This study did not generate new unique reagents.

### Experimental model and subject details

#### Ethics statement

The number of animals and all animal procedures performed in this work were approved by the Institutional Animal Care and Use Committee at the University of California, Davis (IACUC Protocol #21735). Animals were maintained in accordance with the American Association for Accreditation of Laboratory Animal Care guidelines and the 2011 Guide for the Care and Use of Laboratory Animals.^30^ No effort was spared to minimize animal suffering in the course of the experiments reported here.

#### Animals

Fourteen colony-bred Indian-origin rhesus macaques (*Macaca mulatta*) of both sexes were employed in this study. Animals were divided into two main age ranges: young adults (4–12 years old, n = 7) and aged subjects (18–24 years old, n = 7). The young adult group was subdivided into three subgroups: non-infected non-diabetic (n = 2), non-infected type 2 diabetic (n = 1), and infected non-type 2 diabetic (n = 4). The aged adult group was also subdivided into three groups: non-infected non-diabetic (n = 2), non-infected type 2 diabetic (n = 1), and infected type 2 diabetic (n = 4). Diabetic animals were identified based on glycosylated hemoglobin values and continued to receive insulin therapy in addition to oral glucose control medications during the study period. Both sex distribution and the assignment of animals to each group were driven partially by colony constraints, particularly in regard to spontaneous type 2 diabetic animals, which are rare occurrences in the CNPRC colony. Detailed information for each animal’s chronic illness, as well as COVID-19 developed pathology, are provided in Table S1 and Data S1.

Prior to the study initiation, animals were confirmed to be seronegative for SARS-CoV-2 and were kept in a special barrier room. Shortly before inoculation, animals were transferred to an animal biosafety level 3 (ABSL-3) facility, where they were kept individually in indoor stainless-steel cages (Lab Product, Inc.) following national standards of sizing. Animals were exposed to a 12:12-h light/dark cycle, controlled temperature between 64 and 84°F, and controlled humidity between 30 and 70%. Animals had *ad libitum* access to water and received commercial chow (high protein diet; Ralston Purina Co.) and fresh produce supplements.

#### Virus inoculation

Animals assigned to infected subgroups were inoculated with a total of 2.5mL (2.5 × 10^6^ PFU) of SARS-CoV-2 2019-nCoV/USA-WA1/2020 (NR-52352, Lot/Batch #70033952; BEI Resources). Two milliliters of viral stock were delivered intratracheally via an 8 Fr feeding tube, and 0.25mL were administered directly into each nostril. The high inoculum dose used in this study has resulted in peak levels of replication within 1–2 days after inoculation as previously described.^13^^,^^14^^,^^15^

#### Clinical observations

A veterinarian blinded to animal group assignments was responsible for daily cage-side clinical monitoring, including records of responsiveness, discharge, respiratory rate and character, evidence of coughing and sneezing, appetite, and stool quality. Additional clinical assessments, such as rectal temperature, respiration, spO2, heart rate, and skin turgor were performed when animals had to be sedated for procedures. Animals were sedated with ketamine HCl (10mg/kg IM) for the clinical assessment, with dexmedetomidine (15 μg/kg IM) and midazolam (0.25–0.5mg/kg IM) administered after assessment to facilitate sampling, as needed. A summary of clinical findings is presented in Table S1 and Data S1, and in-depth clinical assessment scores for these animals can be found in.^13^^,^^14^^,^^15^

### Method details

#### Euthanasia *and* sample collection

On day 7 post-infection, animals were euthanized with an excess of pentobarbital and a full necropsy was performed under BSL-3 conditions. Following a full craniotomy, brains were removed, cut into 6mm blocks, and immersion-fixed in 10% formalin for 72 h at 4°C. Following fixation, brain blocks were cut into 50μm coronal sections using a vibratome and stored in PBS with 0.1% Azide to prevent microbial and fungal growth (Figure S1).

#### Immunohistochemistry

Free-floating 50μm-thick sections were treated for IHC as previously described.^16^^,^^17^ Briefly, sections were treated with antigen retrieval solution (Cat. #S1700; Wako) at 60°C for 30 min and then washed with PBS. Sections were then incubated in a permeabilizing blocking solution (5% bovine serum albumin, 5% normal goat serum, 5% normal donkey serum, 0.3% Triton X-100 in PBS) at room temperature for 2 h with gentle agitation. Detection of targets was performed by incubating sections in a cocktail of primary antibodies for 48 h at 4°C under agitation. The tissue was then washed with PBS and incubated with Alexa Fluor-conjugated secondary antibodies (1:500, Invitrogen) for 2 h at room temperature under agitation. After additional rinses in PBS, sections were counterstained with 0.5μg/mL of DAPI (Cat #D1306; Invitrogen) and treated with autofluorescence eliminator reagent (Cat. #2160; EMD Millipore) prior to being mounted on slides with ProLong Gold Antifade Mountant (Cat. #P36930; Invitrogen).

Primary antibody cocktails were comprised of antibodies generated in different species/cell lines or different isotypes of mouse IgG. In some instances, multiple antibodies for the same target were used to expand the number of antibody combinations that could be employed. Regardless, comparative quantitative analyses were always performed with the same antibody, ensuring consistency within analyses. A list of primary antibodies used in this study and their respective dilutions can be found in Figure S1.

#### Microscopy and image analysis

All images were acquired in 3D (z stack between 25 and 50μm depending on the experiment) using an LSM800 confocal laser scanning head equipped with two GaAsP photomultiplier tubes mounted on an AxioImager Z1 upright microscope (Carl Zeiss). For viral particle detection and quantification, super-resolution microscopy was achieved using an Airyscan (Carl Zeiss) 32-channel area detector to collect pinhole-plane images at every scan position, resulting in a significantly reduced signal-to-noise ratio. All samples were coded, and image acquisition and analysis were performed by researchers blinded to the experimental groups. Photomicrographs were assembled into figures using the Photoshop 2022 software (Adobe) and illustration were prepared using the Illustrator 2022 software (Adobe).

#### SARS-CoV-2 intracellular N protein volume

To identify the profile of infected cells, a quadruple staining combining the neuronal marker NeuN, the astrocytic marker GFAP, the microglial marker Iba1, and the SARS-CoV-2 N protein was performed targeting layers I-III of the piriform cortex of young and aged animals. Additional analysis was performed in the same region of non-infected age-matched controls to ensure the specificity of the viral staining observed. Four young and four aged, infected animals were analyzed. Twenty randomly chosen fields were acquired with a 63X objective and deconvolved after Airyscan acquisition. Images were then exported and analyzed in 3D using the Imaris software 9.8 (Bitplane). Internalized N protein volume was then calculated in 3D and divided by the total 3D volume obtained for each infected cell type analyzed.

#### Intraneuronal dsRNA and spike volumes

A quadruple staining for DAPI, the neuronal marker MAP2, and viral markers spike (Spk) protein and dsRNA was performed in the piriform cortex, olfactory tubercle, rostromedial entorhinal cortex (also known as the olfactory part), and medial orbitofrontal cortex (a14). These areas were selected based on preliminary distribution studies of viral proteins and the well-established connectivity between these areas through the olfactory circuit.^31^^,^^32^ For each of the olfactory regions analyzed, 10–15 63X Airyscan images were obtained and deconvoluted for 3D volume analysis in Imaris. The total internalized dsRNA or spike viral volumes were divided by the total volume of MAP2 to obtain the percentage of total viral internalized particles.

#### SARS-CoV-2 induced neuroinflammation

To investigate microglia and astrocyte local recruitment to the cortical layers of primary olfactory regions, all 14 animals included in this study were analyzed. Two combinations of 4 antibodies were used for this analysis: DAPI + NeuN + Iba1+GFAP and DAPI + NeuN + Iba1+HLA-DR. ROI selection – First, using a 5X objective to acquire images exclusively on the DAPI channel, a tiled image of the entire section was acquired. Using the 5X tiled image, three ROIs were selected based on anatomical landmarks to be imaged with a 20X objective for later quantification. For example, in the piriform cortex: ROI-1 was centered around the frontal lobe piriform cortex, ROI-2 was centered around the junction of the frontal and temporal lobes, and ROI-3 was centered around the temporal lobe piriform. Great care was taken to ensure these three ROIs were anatomically consistent between all sections imaged. All images were exported coded and analyzed by a second researcher blind to the experimental groups in Imaris. The total number of microglia, astrocytes, and neurons was quantified in 3D for each z stack taken.

#### 3D microglial volume and engulfment activity

For microglial engulfment analysis in the primary olfactory cortex, 20 microglia selected in a blinded fashion were selected from the piriform cortex of each animal. For each microglia, a z stack obtained with a 63X objective was collected, and the image was exported to the Imaris software to create a 3D volume surface rendering of each z stack. Engulfment quantification was done similarly to that previously described by us and others.^16^^,^^17^^,^^33^^,^^34^ Briefly, images were acquired and exported in 3D, where PSD95 puncta, a synaptic marker observed in excitatory synapses, was 3D surface rendered using the same parameters for all the animals. To measure the percentage of engulfment, the volume of internalized puncta (μm^3^) was divided by the total volume of microglial cells (μm^3^).

### Quantification and statistical analysis

All analyses were performed in GraphPad Prism 9 (GraphPad), and datasets were assessed for normality parameters prior to significance determination. Values are expressed as means ± standard error of the mean in the text and in the figures. Statistical tests and p values are indicated in the main text or in the figure legend.

## Data Availability

Microscopy data reported in this paper will be shared by the lead contact upon request, including original unmodified photomicrographs (.czi) and Imaris three-dimensional reconstruction files (.ims). This paper does not report any original code.

## Supplemental information

Document S1. Figures S1–S8, Table S1, and Data S1

Document S2. Article plus supplemental information
