# Supplementary material for: SARS-CoV-2 infects neurons and induces neuroinflammation in a non-human primate model of COVID-19
Source: Cell Rep. 2022 Oct 12;41(5):111573. doi: 10.1016/j.celrep.2022.111573 (PMC9554328; doi:10.1016/j.celrep.2022.111573)
Supplement: Document S1. Figures S1–S8, Table S1, and Data S1 [file mmc1.pdf]

Cell Reports, Volume *41*

Supplemental information

SARS-CoV-2 infects neurons

and induces neuroinflammation

in a non-human primate model of COVID-19

Danielle Beckman, Alyssa Bonillas, Giovanne B. Diniz, Sean Ott, Jamin W. Roh, Sonny R. Elizaldi, Brian A. Schmidt, Rebecca L. Sammak, Koen K.A. Van Rompay, Smita S. Iyer, and John H. Morrison

A Methodological design

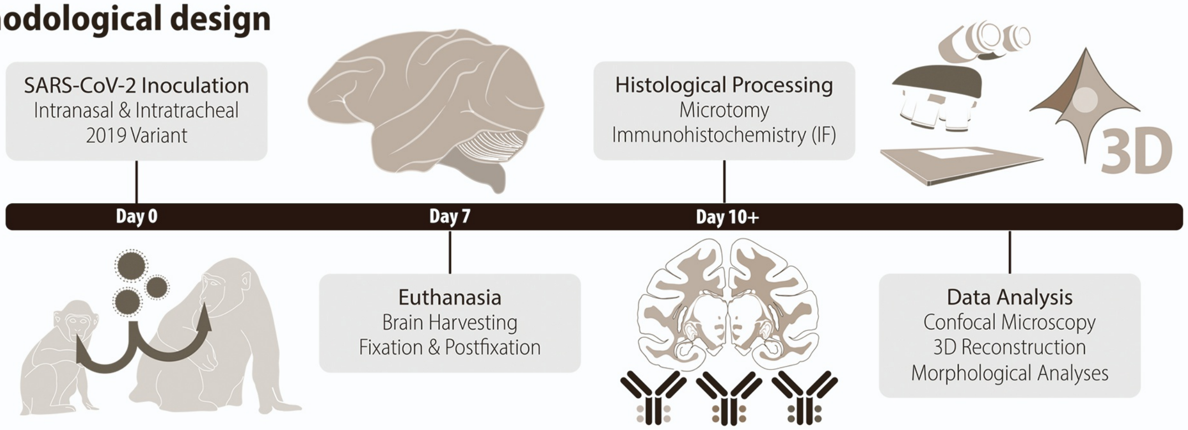

B Major cell types and targets

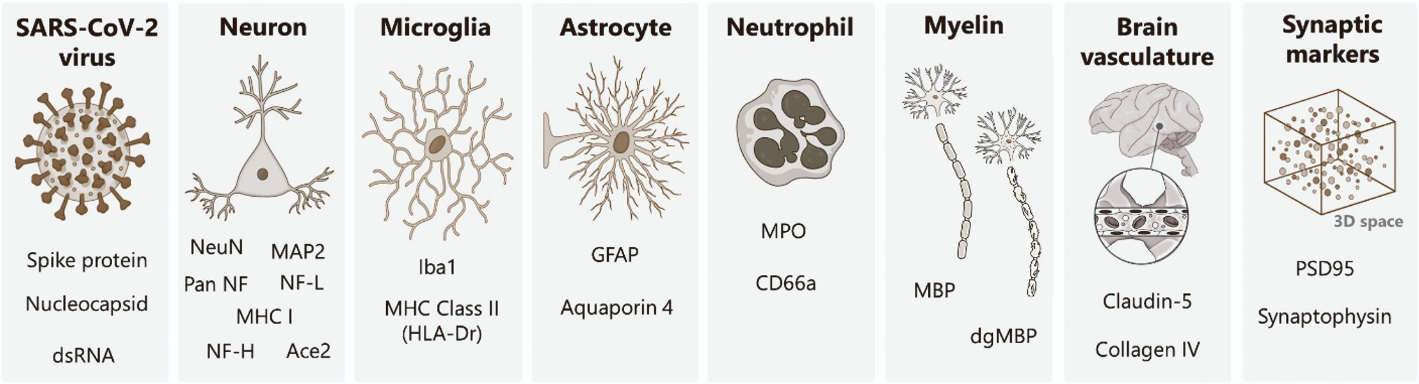

C List of primary antibodies used in this study

Target Protein	Species	Clonality	Isotype	Dilution	Vendor and product code	#RRID
Angiotensin-Converting Enzyme 2 (ACE2)	Rabbit	Polyclonal	IgG	1:100-1:400	Abcam ab15348	AB_301861
Angiotensin-Converting Enzyme 2 (ACE2)	Goat	Polyclonal	IgG	1:50-1:100	R&D Systems AF933	AB_355722
Aquaporin 4 (AQ4)	Guinea Pig	Polyclonal	IgG	1:500	Synaptic Systems 429004	AB_2802156
Cluster of Differentiation 66a (CD66a)	Rat	Monoclonal	IgG2a	1:500	Bio-Rad MCA1147G	AB_323396
Claudin-5	Rabbit	Monoclonal	IgG	1:500	Abcam ab131259	AB_11157940
Collagen Type IV (Collagen IV)	Goat	Polyclonal	IgG	1:100	Millipore AB769	AB_11210995
Degraded Myelin Basic Protein (dgMBP)	Rabbit	Polyclonal	IgG	1:500	US Biological M9758-04	-
Double-stranded RNA (dsRNA)	Mouse	Monoclonal	IgG2a	1:400	SciCons 10010200	AB_2651015
Glial Fibrillary Acidic Protein (GFAP)	Chicken	Polyclonal	IgY	1:1000	Abcam ab4674	AB_304558
Ionized calcium binding adaptor molecule 1 (Iba1)	Rabbit	Polyclonal	IgG	1:500	Fujifilm Wako 019-19741	AB_839504
Ionized calcium binding adaptor molecule 1 (Iba1)	Mouse	Monoclonal	IgG2b	1:500	Fujifilm Wako NCNP24	AB_2811160
Microtubule-Associated Protein 2 (MAP2)	Guinea Pig	Polyclonal	IgG	1:1000	Synaptic Systems 188004	AB_2138181
Major Histocompatibility Complex Class I (HLA-E)	Rabbit	Monoclonal	IgG	1:200	Novus Bio NBP2-66946	AB_2809803
Major Histocompatibility Complex Class II (HLA-DR)	Mouse	Monoclonal	IgG2b	1:200	BioLegend 327002	AB_893582
Major Histocompatibility Complex Class II (HLA-DR)	Mouse	Monoclonal	IgG2b	1:200	Thermo Fisher MA5-11966	AB_10979984
Myelin Basic Protein (MBP)	Human	Monoclonal	IgG1	1:500	Abcam ab209328	AB_2818988
Myeloperoxidase (MPO)	Mouse	Monoclonal	IgG1	1:500	Bio-Rad MCA1757	AB_2146467
Neuronal Nuclear Protein (NeuN)	Guinea Pig	Polyclonal	IgG	1:500	Synaptic Systems 266004	AB_2619988
Neuronal Nuclear Protein (NeuN)	Mouse	Monoclonal	IgG1	1:500	Millipore MAB377	AB_2298772
Neurofilament- Heavy (NF-H)	Mouse	Monoclonal	IgG1	1:200	BioLegend 801701	AB_2715852
Neurofilament- Heavy (NF-H)	Chicken	Polyclonal	IgY	1:500	Abcam ab4680	AB_304560
Neurofilament- Light (NF-L)	Mouse	Monoclonal	IgG1	1:500	Millipore MAB1615	AB_94285
Pan-Neurofilament (Pan-NF)	Mouse	Monoclonal	IgG1/IgM	1:400	Creative Diagnostic DMAB7133	AB_2391764
Postsynaptic Density Protein (PSD95)	Goat	Polyclonal	IgG	1:500	Abcam ab12093	AB_298846
SARS-CoV-2 Nucleocapsid (N) protein	Mouse	Monoclonal	IgG2b	1:100	Thermo Fisher MA1-7403	AB_1018420
SARS-CoV-2 Spike Protein	Rabbit	Polyclonal	IgG	1:500	Abcam ab272504	AB_2847845
SARS-CoV-2 Spike Protein	Human	Monoclonal	IgG1	1:100	Thermo Fisher 703958	AB_2866477
Synaptophysin 1	Guinea Pig	Polyclonal	IgG	1:500	Synaptic Systems 101004	AB_1210382

#RRID – Research Resource Identifier

Figure S1. Methodological overview of the study, Related to STAR Methods. (A) Schematic representation of the study methodology. Four young healthy (3.5-6 Y.O) and four aged (18-22 Y.O) rhesus monkeys were infected intranasally and intratracheally with SARS-CoV-2 (2019-nCoV/USA-WA1/2020; BEI Resources) at high dose (2.5x106 plaque-forming units [PFU]) and euthanized one week later. In addition, non-infected brains from diabetic and non-diabetic animals were processed in a similar way for the same microscopy analysis. (B) Major targets investigated in this study and the markers used to identify them. (C) List of primary antibodies used in this study.

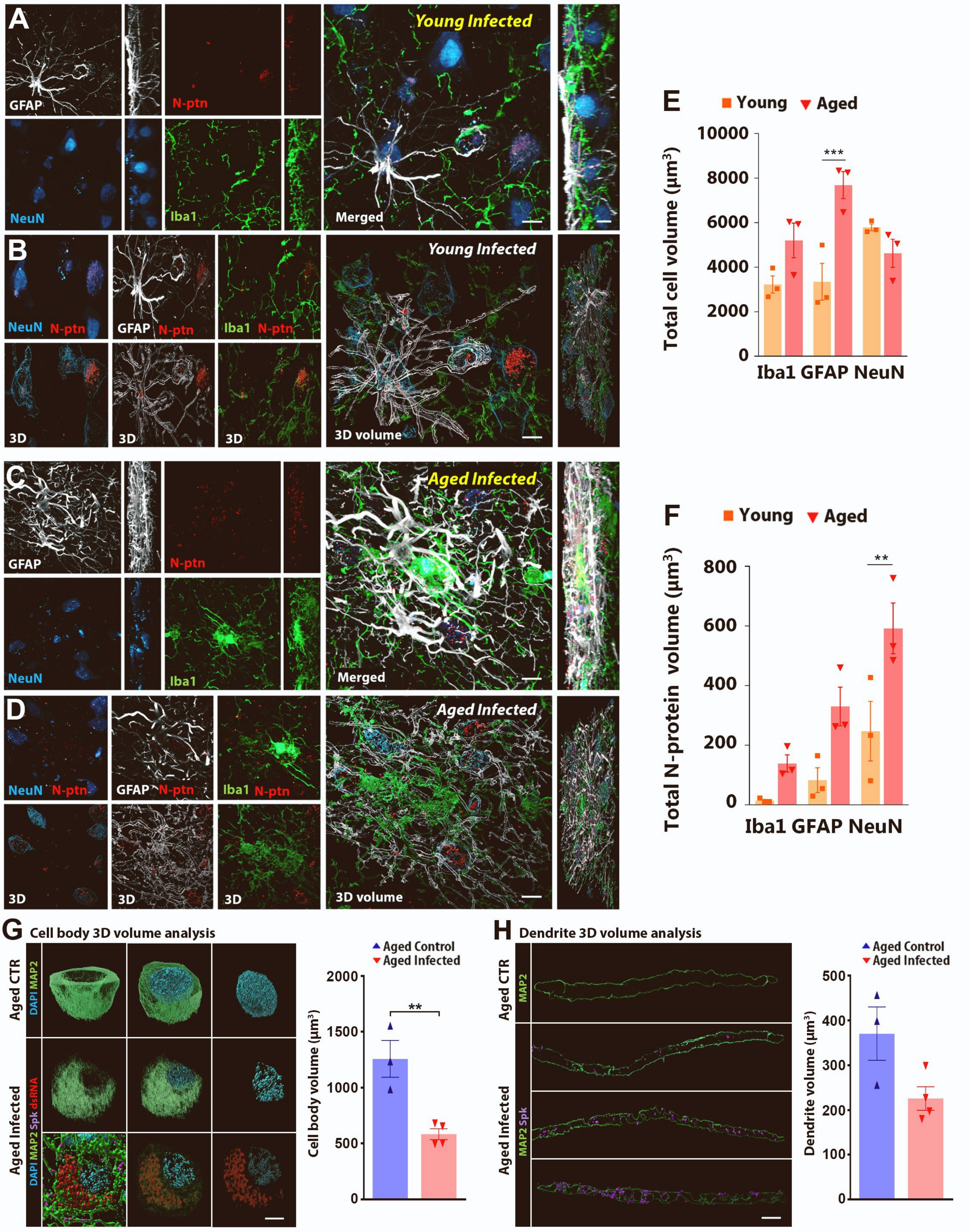

A
GFAP
N-ptn
NeuN
Iba1
Young Infected
Merged
B
NeuN N-ptn
GFAP N-ptn
Iba1 N-ptn
3D
Young Infected
3D volume
C
Aged Infected
D
Aged Infected
E
Young
Aged
Total cell volume (µm³)
10000
8000
6000
4000
2000
0
***
Iba1 GFAP NeuN
F
Total N-protein volume (µm³)
800
600
400
200
**
G
Cell body 3D volume analysis
Aged CTR
DAPI MAP2
Aged Infected
DAPI MAP2 Spk dsRNA
Aged Control
Aged Infected
Cell body volume (µm³)
2000
1500
1000
500
H
Dendrite 3D volume analysis
MAP2
MAP2 Spk
Dendrite volume (µm³)
500
400
300
200
100

Figure S2. Complementary volumetric analyses of neuronal morphology, infection tropism, and neuro-inflammation, Related to Figure 01. (A-F) In young (A, B) and aged (C, D) infected animals, individual and combined channels are shown in the XY and XZ planes. In (B) and (D), detail of the original images and the 3D volume reconstructions used to identify and quantify the cell types infected and the total internalized viral volume. In comparison with young, infected animals, aged-infected monkeys present a significant increase in astrocyte hypertrophy in comparison with the other cell types (E, ***p=0.0009). In contrast, SARS-CoV2 nucleocapsid protein was detected in higher quantities in neurons and significantly in higher levels in aged, infected animals (F, **p= 0.0069). Two-way ANOVA, Sidak's post hoc test. **(G, H)** To better understand the extensive neuronal cell damage observed in the primary olfactory cortex following SARS-CoV-2 infection, we developed a protocol to calculate how the virus affects the cell body and dendrites separately. In the cell body of neurons within layers I-III of the piriform cortex, dsRNA vesicles were found associated with degraded neuron-specific microtubule stabilizer MAP2, and a reduced cell body volume was observed in aged, infected animals in comparison with age-matched controls (G, **p=0.0032). Dendritic beading, an early marker of neuronal injury and swelling, was observed within neuronal dendrites expressing high levels of spike protein and is correlated with decreased dendritic total volume (H, *p= 0.0571). Unpaired t-test. Data are represented as mean ± SEM. Scale bar: 50μm (A-D), 5μm (G, H).

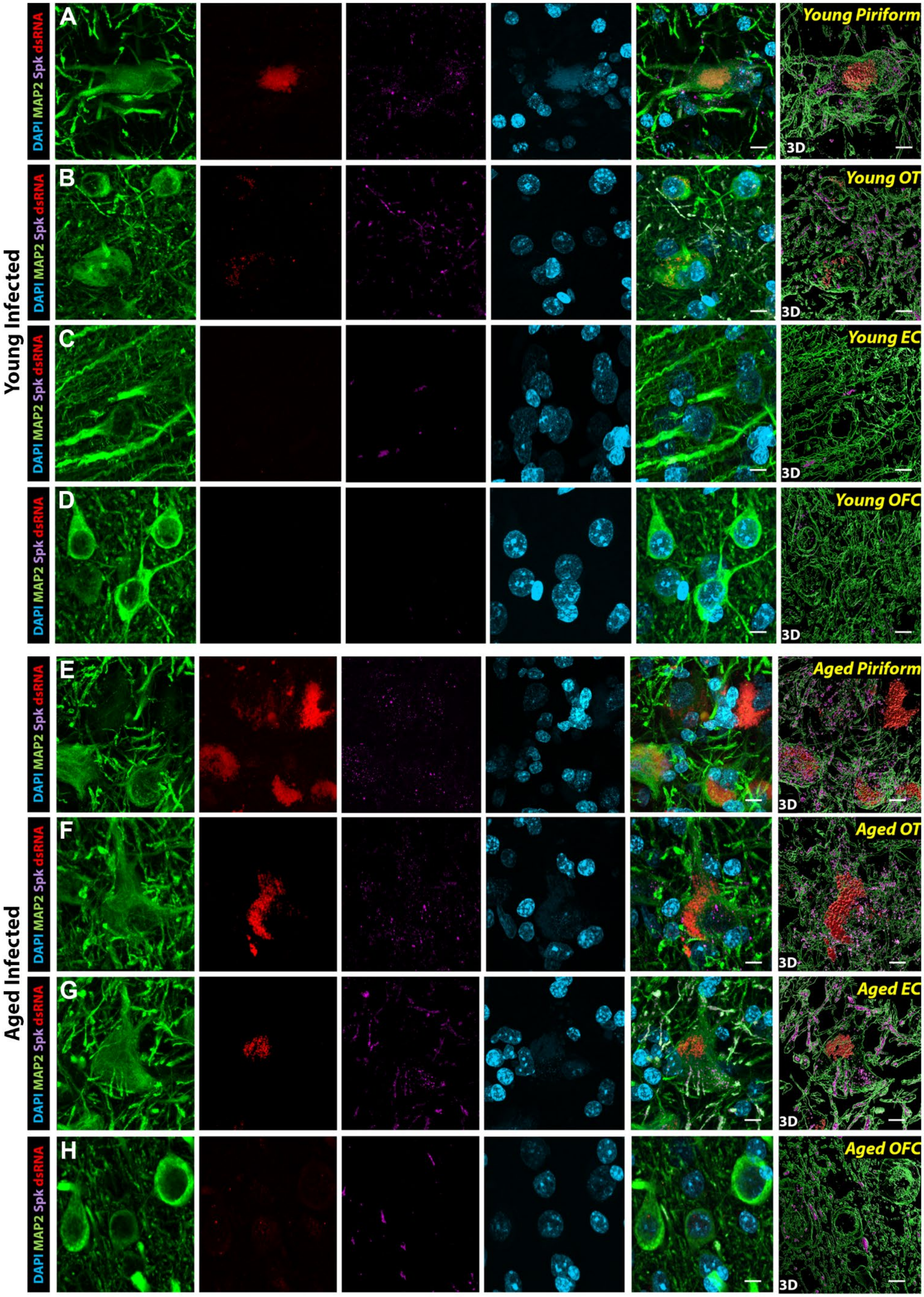
Young Infected
Aged Infected
A
B
C
D
E
F
G
H
DAPI MAP2 Spk dsRNA
Young Piriform
Young OT
Young EC
Young OFC
Aged Piriform
Aged OT
Aged EC
Aged OFC
3D

Figure S3. Viral RNA and SARS-CoV2 spike protein in the olfactory cortex of infected animals, Related to Figure 01. (A-D) Individual and merged representative micrographs and 3D volume reconstruction of olfactory regions of young, infected animals: piriform (A), olfactory tubercle (B), entorhinal cortex (C), and orbitofrontal cortex (D). Double-stranded RNA and SARS-CoV2 spike protein are strongly detected in primary but not secondary olfactory regions of young, infected monkeys. **(E-H)** Individual and merged representative micrographs and 3D volume reconstruction of olfactory regions of aged, infected animals: piriform (E), olfactory tubercle (F), entorhinal cortex (G), and orbitofrontal cortex (H). In comparison to young animals, aged-infected animals present considerably more neuronal infection and SARS-CoV2 viral protein spread reaching the OFC, presumably through neuronal connections. Scale bar: 50μm.

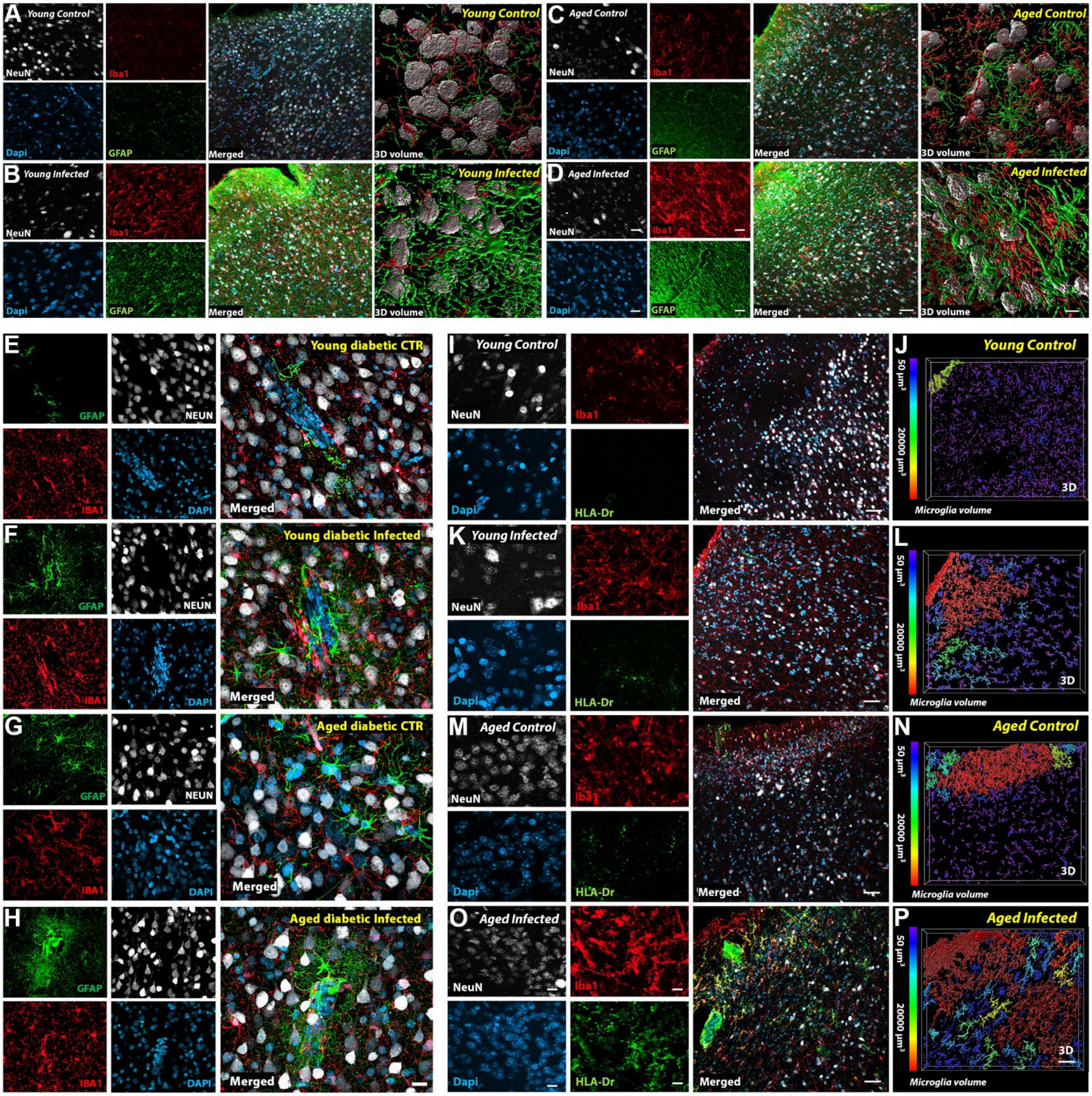

Figure S4. Glial and vascular disruption induced by SARS-CoV-2 infection, Related to Figure 02. (A-D) Each panel shows representative micrographs and 3D volume reconstructions of the experimental groups: (A) young control, (B) young infected, (C) aged control, and (D) aged infected monkeys. The individual and merged micrographs show astrocytes (GFAP) and microglia (Iba1) interaction with neurons (NeuN). Detailed 3D reconstruction analysis highlights the glia morphological profile change induced by the SARS-CoV2 infection in the brain. **(E-H)** Individual channels and merged images of the piriform cortex from young diabetic CTR (E), young SARS-CoV2 infected (F), aged diabetic CTR (G), and aged diabetic infected (H) monkeys. Morphological alterations in astrocytes (GFAP) and microglia (Iba1) were associated with the diabetes condition but worsened following SARS-CoV2 infection. **(I-P)** The human leukocyte antigen (HLA, type D.R.) is a major histocompatibility complex class II (MHCII) receptor expressed by the microglial population upon disturbances in the immunological homeostasis in the CNS, participating in the recruitment of CNS-infiltrating T cells. As shown in the representative individual and merged images of each group (I, K, M, O), there is an increased expression of this receptor in the aged-infected animals in comparison with the other groups. Notably, a heat-map representation of the microglia volume profile observed in the olfactory region (J, L, N, P) shows a progressive increase in the number of individual cells presenting

altered volume, a marker of cellular activation and neuronal damage (heat-map shows a range of microglia volume distribution from 50 μm^3/blue to 2000 μm^3/red). Scale bar: 100μm (A-D photomicrographs; I-P), 50μm (A-D 3D volumes), 45μm (E-H).

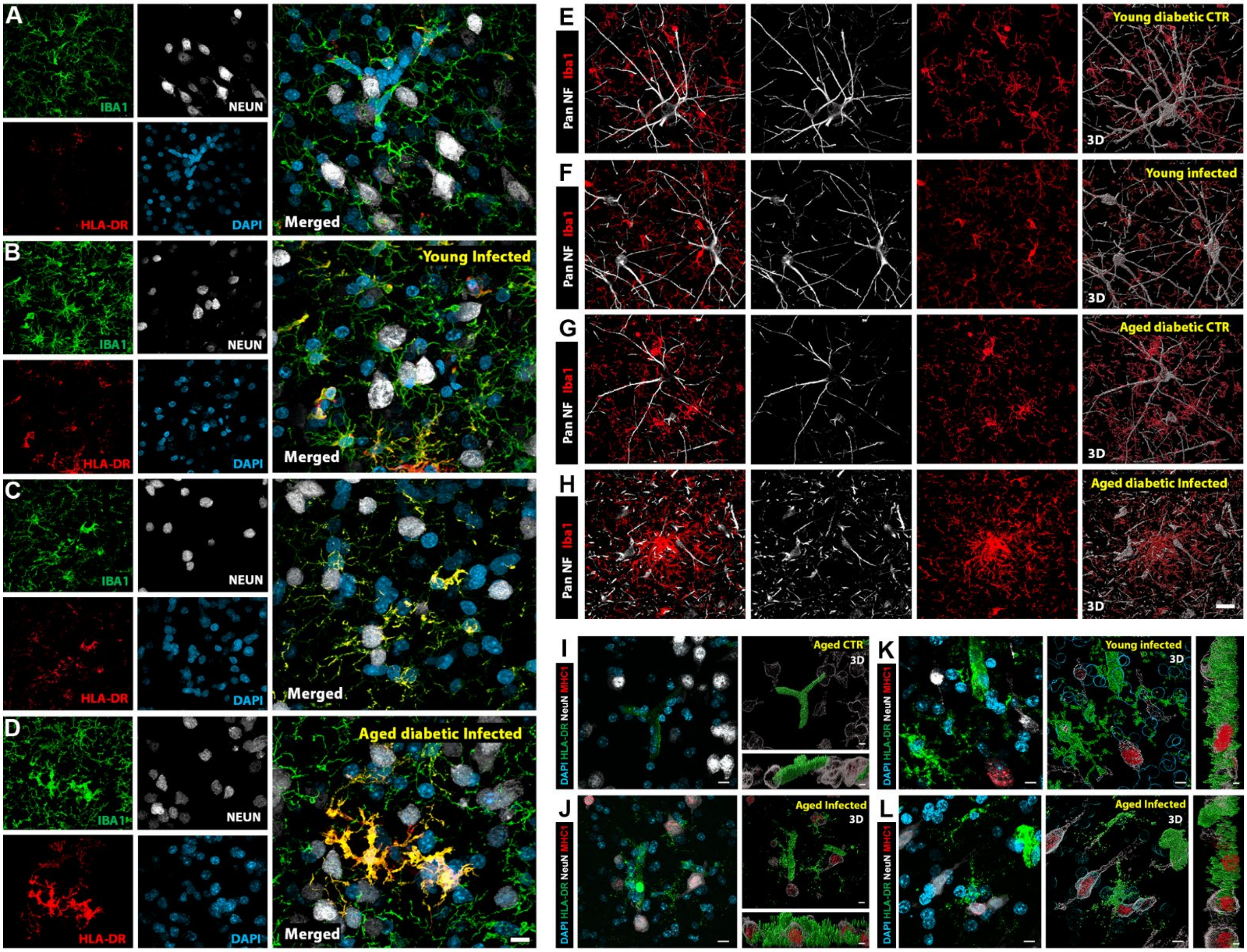

Figure S5. SARS-CoV-2 neuroinfection is associated with MHC Class I and II upregulation and neuron-microglia interaction disruption, Related to Figure 02. (A-D) Representative images from young diabetic CTR (A), young SARS-CoV2 infected (B), aged diabetic CTR (C), and aged diabetic infected (D) monkeys highlight the increased microglial expression of HLA-DR observed in both SARS-CoV2 infected groups. Increased HLA-DR expression was also observed in the aged diabetic CTR animal but was worsened by the viral infection. **(E-H)** Using pan NF as a neuronal marker and Iba1 to label microglia, we applied Airyscan super-resolution microscopy to investigate how diabetes and SARS-CoV2 infection alter neuron-microglia dynamics. In young diabetic CTR (E) and young infected (F) animals, no substantial alterations were found in the primary olfactory cortex. On the other hand, a more activated microglial profile was observed in the aged diabetic CTR (G) and was notably worsened by SARS-CoV2 infection, as shown in the representative micrograph from an aged diabetic infected animal (H). **(I-L)** In aged control monkeys, HLA-DR is usually found in low quantities surrounding intact blood vessels, in physiological balance with the neuronal and glial population. On the other hand, MHC1 is not generally expressed in healthy adult brains, and no expression was detected in the aged CTR brain (I). Following the viral infection in aged animals, microglia cells expressing the MHC class II receptor HLA-DR are recruited locally and found in association with altered blood vessels and MHC1+ neurons (J). Both classes of proteins are essential for the recognition of cytotoxic T cells and markers of T cell infiltration in the brain. To further understand the role of the antigen presentation by MHC proteins in the brain following SARS-CoV-2 infection in young and aged infected animals, we further analyzed the expression of both adaptive immunity markers in the olfactory cortex using 3D volumetric reconstruction. As shown in representative images in (K) and (L), in both the XY and XZ planes, neurons MHC1+ and microglia HLA-DR+ in association with disruptive blood vessels are found in both infected groups, but with higher frequency in the aged, infected animals. Scale bar: 20µm (A-D), 10µm (E-L photomicrographs), 5µm (I-L 3D Volumes).

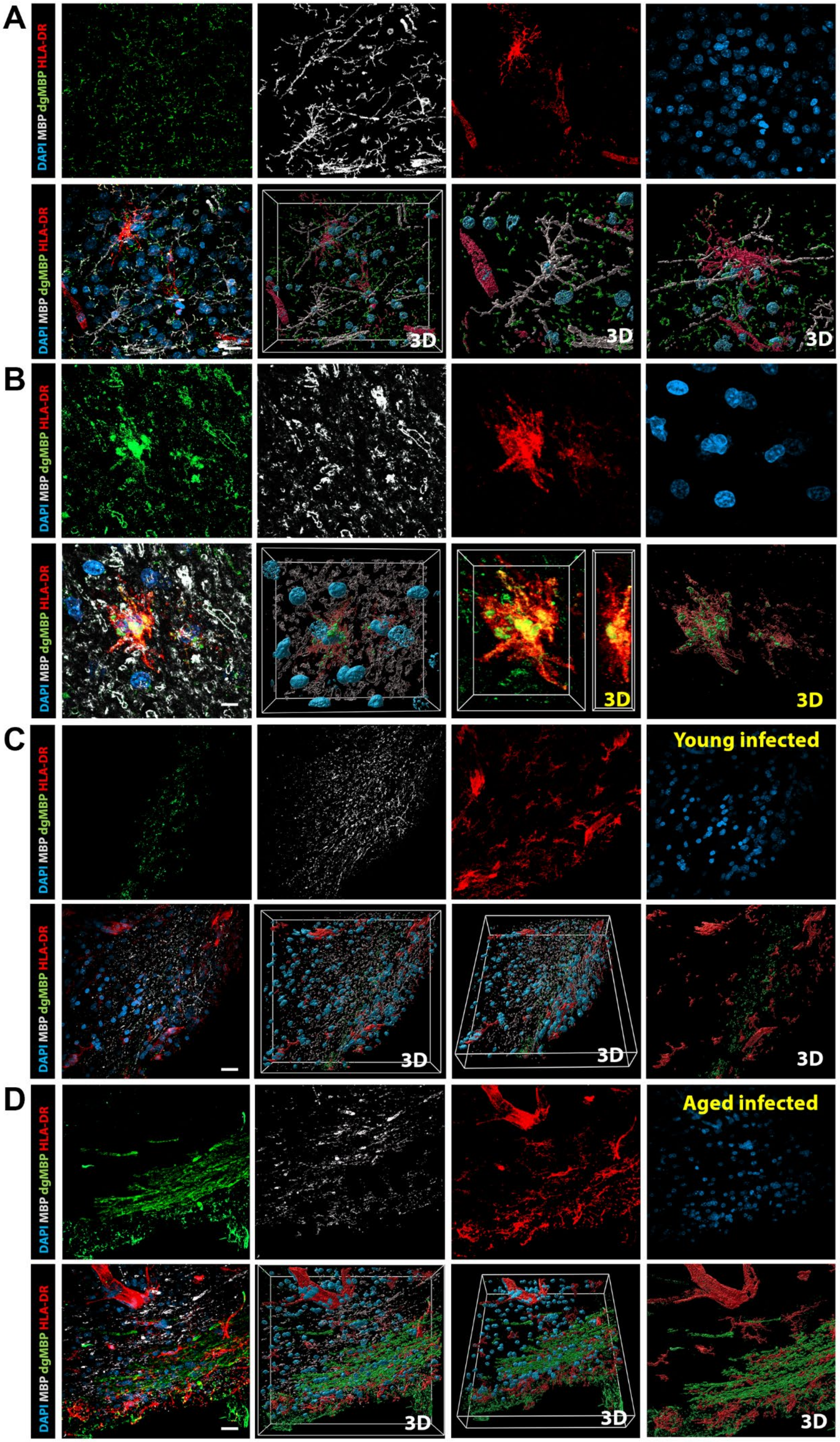
A
DAPI MBP dgMBP HLA-DR
3D
B
C
Young infected
D
Aged infected

Figure S6. Increased expression of degraded myelin and HLA-DR expression found in SARS-CoV2 infected animals, Related to Figure 02. A combination of antibodies targeting normal (MBP, white) and degraded myelin (dgMBP, green) were used with HLA-DR (microglia and blood vessels) and DAPI. Three-dimensional analyses in the piriform cortex show increased expression of dgMBP at the expense of the normal MBP, associated with increased HLA-DR expression (A, B). Comparison of the piriform cortex from young and aged infected animals highlights an increased presence of dgMBP, associated with increased expression of HLA-DR (C, D). Scale bar: 50µm.

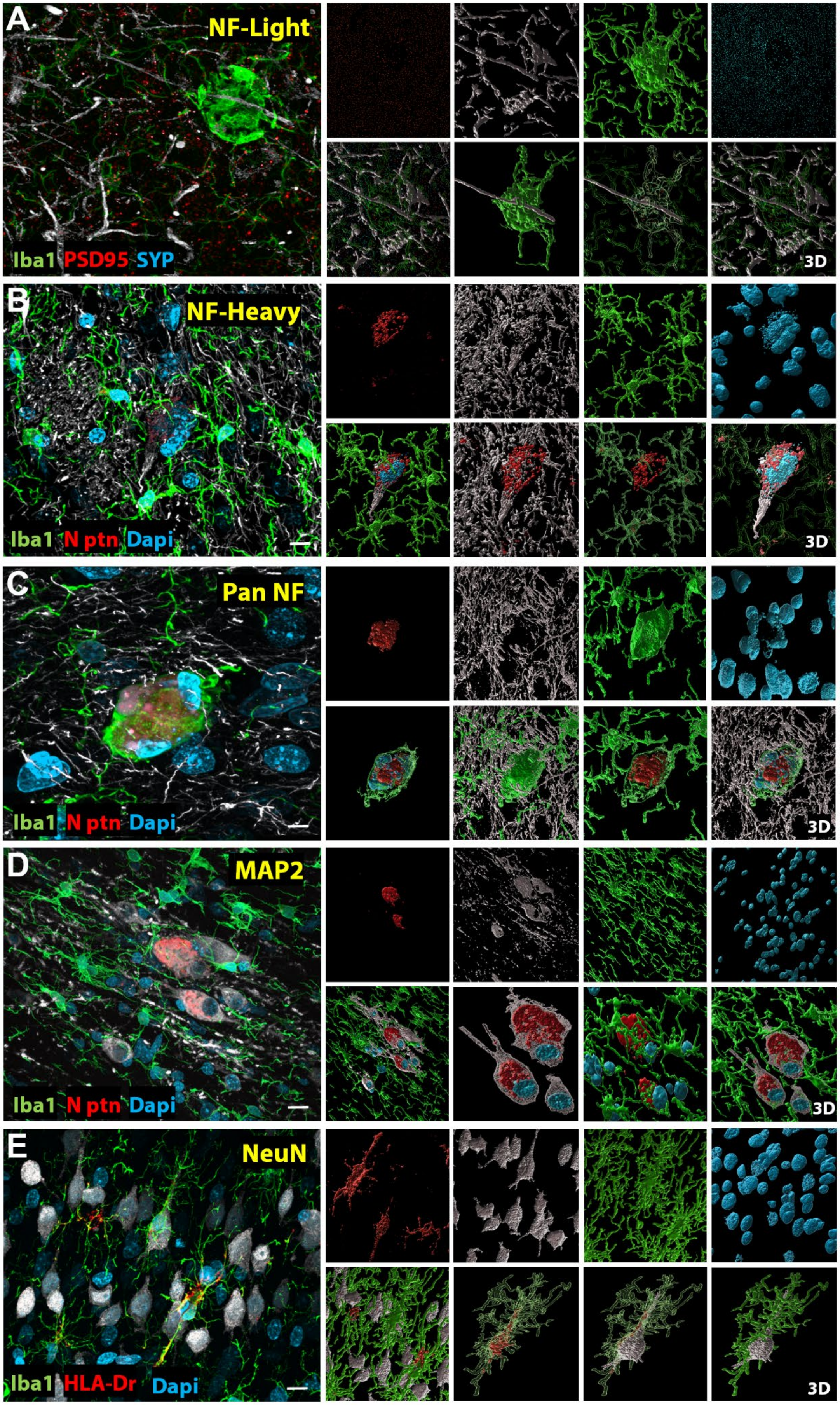

A
NF-Light
Iba1 PSD95 SYP
3D
B
NF-Heavy
Iba1 N ptn Dapi
3D
C
Pan NF
Iba1 N ptn Dapi
3D
D
MAP2
Iba1 N ptn Dapi
3D
E
NeuN
Iba1 HLA-Dr Dapi
3D

Figure S7. SARS-CoV-2 presence in the brain distorts regular neuron-microglia interaction, Related to Figure 02. Multilabel fluorescence microscopy was performed targeting several neural proteins involved in synaptic transmission and neuron-glia interaction. For this analysis, adjacent stereological sections (every 50 μm) of the primary olfactory cortex from infected monkeys were used. As shown in (A), clusters of microglia (Iba1) are actively interacting with dendrites (neurofilament light, NF-L), and the presence of presynaptic marker synaptophysin (SYP) and postsynaptic marker (PSD95), were observed in abundance within reactive microglia. Interestingly, as demonstrated in (B), neurons expressing the SARS-CoV-2 nucleocapsid protein (N ptn) still present a more preserved neurofilament-heavy (NF-H) structure. (C) Utilizing pan NF marker combined with Iba1, we were able to detect entire neurons expressing N ptn being engulfed by reactive microglia. The reactive profile of the microglia interacting with neurons was confirmed by combining microglia general marker Iba1 and activated microglia marker HLA-DR (D). Scale bar: 50 μm.

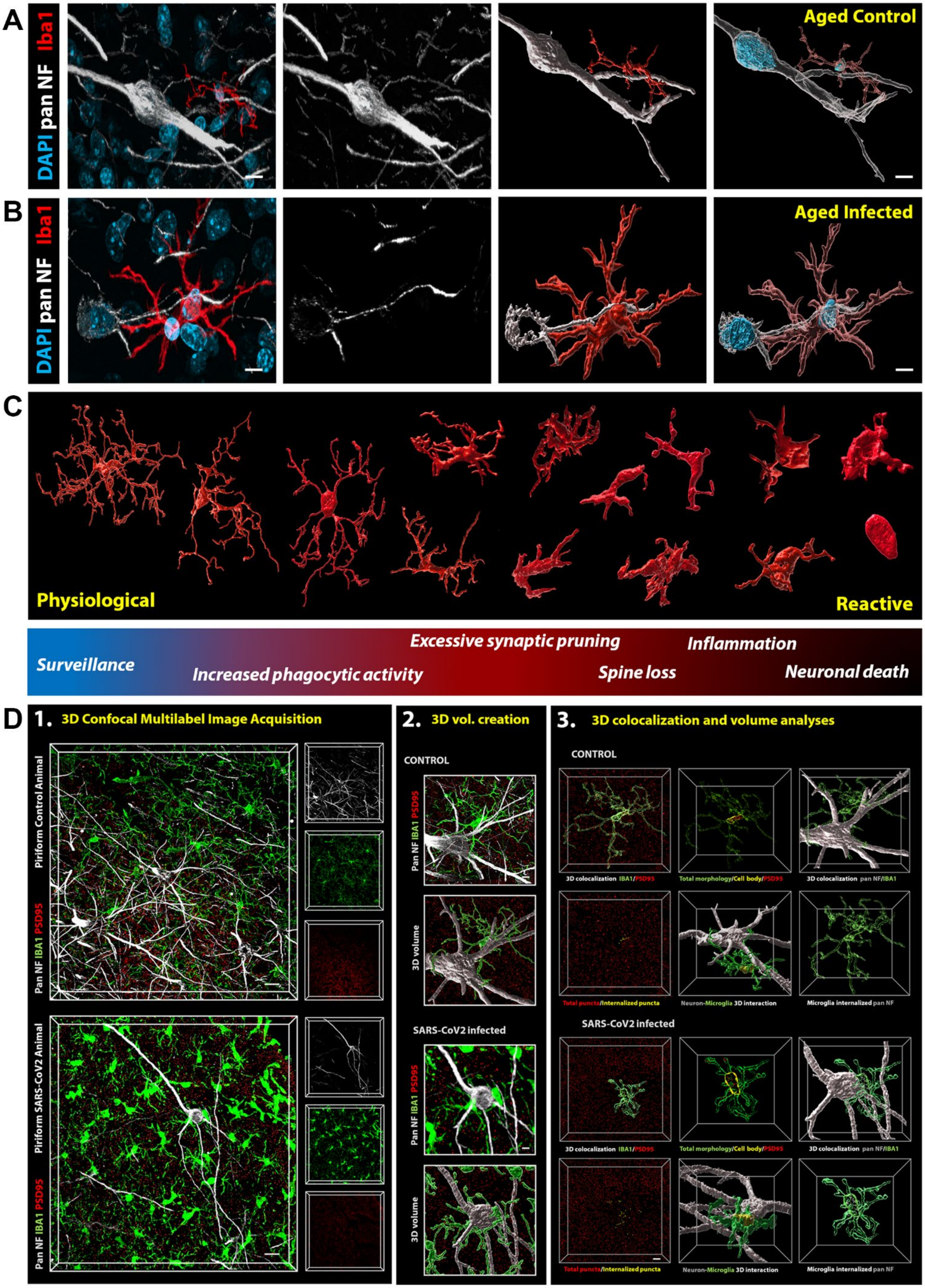
A
DAPI pan NF Iba1
Aged Control
B
DAPI pan NF Iba1
Aged Infected
C
Physiological
Reactive
Surveillance
Increased phagocytic activity
Excessive synaptic pruning
Spine loss
Inflammation
Neuronal death
D
1. 3D Confocal Multilabel Image Acquisition
Piriform Control Animal
Pan NF IBA1 PSD95
Piriform SARS-CoV2 Animal
Pan NF IBA1 PSD95
2. 3D vol. creation
CONTROL
Pan NF IBA1 PSD95
3D volume
SARS-CoV2 infected
Pan NF IBA1 PSD95
3D volume
3. 3D colocalization and volume analyses
CONTROL
3D colocalization IBA1/PSD95
Total morphology/Cell body/PSD95
3D colocalization pan NF/IBA1
Total puncta/Internalized puncta
Neuron-Microglia 3D interaction
Microglia internalized pan NF
SARS-CoV2 infected
3D colocalization IBA1/PSD95
Total morphology/Cell body/PSD95
3D colocalization pan NF/IBA1
Total puncta/Internalized puncta
Neuron-Microglia 3D interaction
Microglia internalized pan NF

Figure S8. Overview of the super-resolution Airyscan microscopy and 3D Segmentation employed to examine cellular morphology, neuron-microglia interactions, and PSD95 puncta engulfment, Related to STAR Methods. (A-C) Compared with age-matched controls (A), high-resolution microscopy analysis of the primary olfactory cortex of infected animals presents abnormal microglia and fragmented/dying neuron interaction (B). Full detailed 3D reconstruction of microglia shows dynamic changes in this cell type profile, closely related to neuroinflammatory process and neurodegeneration (C). **(D)** To investigate the microglial engulfment of synaptic markers and neuronal proteins, micrographs were first acquired using a 63x objective in a confocal microscope (Step 1). Next, images were exported to Imaris software, and a 3D volume surface was created for each marker analyzed (Step 2). The cell body (yellow) and total volumes (green) for each microglia were calculated, as well as the 3D puncta volume of PSD95 (Step 3). Engulfment activity was measured by quantifying the volume of internalized PSD 95 (µm3) divided by the total volume of the involved microglia cell (μm^3). Scale bar: 5µm (A-C), 50µm (D).

Table S1. Information regarding the animals used in this study, including chronic illness and treatments, Related to the STAR methods.

Group	Animal number	Sex	Age	Chronic Illness
Young Control	MMU-1	Female	6	Endometriosis
Young Control	MMU-12	Female	4	None
Young Control	MMU-13	Male	12	Diabetes, cystitis; Concurrent medication: Lantus (insulin glargine)
Young infected	MMU-6	Female	4	None
Young infected	MMU-3	Female	5	Endometriosis
Young infected	MMU-11	Male	4	None
Young infected	MMU-5	Female	6	Endometriosis
Aged Control	MMU-8	Female	19	Cecal Adenocarcinoma
Aged Control	MMU-10	Female	21	Hepatic Amyloidosis, Endometriosis
Aged Control	MMU-14	Male	23	Diabetes, obesity and suspected tract infection. Concurrent medication: Tresiba (insulin deglutec)
Aged infected	MMU-2	Female	20	Diabetes, gastrointestinal adenocarcinoma. Concurrent medication: Tresiba (insulin degludec).
Aged infected	MMU-4	Female	24	Diabetes, hypertension. Concurrent medication: Lantus (insulin glargine), pioglitazone.
Aged infected	MMU-7	Female	22	Diabetes, endometriosis, diverticulosis. Concurrent medication: Tresiba (insulin deglutec)
Aged infected	MMU-9	Female	18	Diabetes, Hypertension. Concurrent medication: Tresiba (insulin degludec).

Animal number	Days after infection	MMU-6	MMU-3	MMU-11	MMU-5	MMU-2	MMU-4	MMU-7	MMU-9
Body Weight (kg)	-2	5.72	9.40	8.81	4.36	8.13	6.40	5.08	7.25
	0	5.04	9.14	7.01	4.00	8.04	6.56	5.16	7.91
	2	6.46	10.49	6.78	4.82	7.56	5.90	4.92	7.03
	4	5.98	10.26	6.92	5.12	7.45	6.14	5.03	6.99
	6	6.14	9.56	7.21	4.98	7.88	6.09	5.77	7.82
Rectal temperature (°C)	-2	100.4	100.2	101.6	99.9	99.1	100.6	99.6	100.6
	0	99.7	101.1	102.1	100.6	100.3	99.9	101.9	101.6
	2	100.7	99.7	103.3	103.2	101.9	101.2	103.4	102.2
	4	100.1	100.4	103.4	102.6	99.9	101.0	102.9	101.4
	6	100.4	100.3	101.7	100.1	101.2	100.9	103.2	101.6
Heart rate (bpm)	-2	144	136	165	121	176	151	191	145
	0	164	140	171	123	181	159	169	138
	2	157	134	182	136	166	162	199	147
	4	141	139	169	144	159	158	196	160
	6	151	142	168	135	177	157	197	152
SpO2	-2	97	99	99	99	100	99	100	99
	0	97	100	98	97	99	98	97	99
	2	98	98	97	97	97	98	97	98
	4	96	99	97	98	99	98	98	97
	6	97	100	97	98	99	99	97	99

Data S1 - Detailed measurements related to COVID-19 development in infected monkeys.

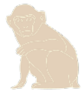

Young infected group

Animal number	Days after infection	Responsiveness/ recumbency	Discharges	Coughing/sneezing	Respiratory character	Respiratory Rate (breaths per min)	Food consumption
MMU-6 Female 4 Y.O	-1	BAR	None	None	WNL	38	2 chow, 0 apple, 0 veg, 12 df, 2 fb, peanuts
	0	BAR, active	None	None	WNL	36	2 chow, 2 apple, 1 veg, 9 df, 2 fb, peanuts
	1	BAR	None	None	WNL, minor shallow breath late	40	2 chow, 2 apple, 1 veg, 9 df, 2 fb, peanuts
	2	BAR	None	None	WNL	32	6 chow, 0 apple, 0 veg, 4 df, 2 fb, peanuts
	3	BAR	None	None	WNL	42	4 chow, 2 apple, 1 veg, 5 df, 1 fb, peanuts
	4	BAR, active	None	None	WNL	36	2 chow, 0 apple, 0 veg, 8 df, 3 fb, peanuts
	5	BAR	None	None	WNL	42	2 chow, 1 apple, 2 veg, 5 df, 4 fb, peanuts
	6	BAR	None	None	WNL	42	0 chow, 2 apple, 2 veg, 8 df, 1 fb, peanuts
	7	BAR, active	None	None	WNL	42	4 chow, 0 apple, 1 veg, 9 df, 3 fb, peanuts
MMU-3 Female 5 Y.O	-1	BAR, lip-smacking	None	None	WNL	42	4 chow, 1 apple, 2 veg, 15 df, 2 fb, peanuts
	0	BAR	None	None	WNL	40	2 chow, 2 apple, 5 df, 1 fb, peanuts
	1	BAR, timid	None	None	WNL	36	4 chow, 4 apple, 2 veg, 2 df, 4 fb, peanuts
	2	BAR	None	None	WNL	36	2 chow, 2 apple, 3 veg, 9 df, 3 fb, peanuts
	3	BAR, timid	None	None	WNL, quick shallow breath all day	42	3 chow, 1 apple, 2 veg, 5 df, 2 fb, 1 banana
	4	BAR	None	None	WNL	36	4 chow, 1 apple, 6 df, 6 fb, peanuts
	5	BAR, timid	None	None	WNL	36	2 chow, 1 apple, 2 veg, 12 df, 2 fb, peanuts
	6	BAR	None	None	WNL	36	4 chow, 2 apple, 3 veg, 7 df, 2 fb, peanuts
	7	BAR	None	None	WNL	42	4 chow, 1 apple, 2 veg, 12 df, 4 fb, peanuts
MMU-11 Male 4 Y.O	-1	BAR	None	None	WNL	48	5 chow, 1 apple, 1 banana, 1 veg, 9 df, 2 fb
	0	BAR, active	None	None	WNL	54	2 chow, 2 apple, 2 veg, 9 df, 2 fb, peanuts
	1	BAR, anxious	None	Single cough AM	WNL, minor ketamine reaction	42	1 chow, 2 apple, 0 veg, 4 df, 6 fb, peanuts
	2	BAR	Mild discharges	A couple of coughs AM	WNL, minor shallow breath	48	4 chow, 1 apple, 2 veg, 7 df, 8 fb, peanuts
	3	BAR, anxious	Mild dried discharges on both nares	Cough with sneeze heard twice	WNL, mild nasal flaring	54	3 chow, 3 apple, 2 veg, 9 df, 4 fb, 1 banana
	4	BAR	Mild discharge with later sneeze	Minor cough and sneezing all day	WNL, mild nasal flaring all day	48	2 chow, 2 apple, 2 veg, 4 df, 8 fb, peanuts
	5	BAR	None	Dry cough during PM	WNL	42	0 chow, 4 apple, 4 veg, 10 df, peanuts
	6	BAR, active	None	None	WNL	48	2 chow, 3 apple, 6 veg, 7 df, peanuts
	7	BAR, active	None	None	WNL	50	5 chow, 2 apple, 3 veg, 8 df, 8 fb, peanuts
MMU-5 Female 6 Y.O	-1	BAR	None	None	WNL	37	4 chow, 2 apple, 2 veg, 9 df, 2 fb, peanuts
	0	BAR, timid	None	None	Minor ket. reaction altering breathing	40	4 chow, 0 apple, 0 veg, 15 df, 2 fb, peanuts
	1	BAR, timid	Small amount bilateral	None	WNL, intermittent apneustic pattern	44	4 chow, 1 apple, 1 veg, 10 df, 1 fb, peanuts
	2	BAR	None initially, later clear R-side	Later cough at PM	WNL, quick shallow breath	48	2 chow, 1/2 apple, 3 veg, 11 df, 6 fb, peanuts
	3	BAR	Small amount bilateral	Minor dry cough all day	WNL, quick shallow breath	50	4 chow, 1 apple, 2 veg, 9 df, 4 fb, 1/2 banana
	4	BAR, active	Mild dried discharges	Minor cough all day	WNL	48	2 chow, 2 apple, 2 veg, 4 df, 8 fb, peanuts
	5	BAR	Mild dried discharges	Vet heard sneezing twice	WNL	42	2 chow, 1/2 apple, 3 veg, 11 df, 7 fb, peanuts
	6	BAR, pacing	None	Single cough AM	WNL	44	4 chow, 2 apple, 2 veg, 10 df,6 fb, peanuts
	7	BAR	None	None	WNL	46	3 chow, 1/2 apple, 1 veg, 6 df, 4 fb, peanuts

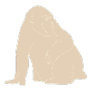

Aged infected group

Animal number	Days after infection	Responsiveness/ recumbency	Discharges	Coughing/sneezing	Respiratory character	Respiratory Rate (breaths per min)	Food consumption
MMU-2 Female 20 Y.O	-1	BAR	None	None	WNL	40	4 chow, 1 apple, 3 veg, 5 df, 2 fb, peanuts
	0	BAR, active	None	None	WNL	39	2 chow, 1/2 apple, 1 veg, 6 df, 1 fb, peanuts
	1	BAR, anxious	None	None	WNL	42	1 chow, 1/2 apple, ½ banana,10 df, 1 fb, peanuts
	2	BAR	None	None at Obs, heard sneezing at 9:35 AM	WNL, occasional quick shallow breath	50	3 chow, 2 apple, 2 veg, 2 df, 5 fb, peanuts
	3	BAR, anxious	None at Obs, observed coughing later	None at Obs, cough earlier in front of tech	Mild nasal flaring with each breath	53	3 chow, 1 apple, 6 df, 4 fb, peanuts
	4	BAR	None at Obs, small amount later	Minor cough and sneezing all day	Mild nasal flaring	53	2 chow, 3 apple, 10 df, 5 fb, peanuts
	5	BAR	Mild dried discharges	Single cough at PM	WNL	51	2 chow, 3 apple, 2 veg, 8 df, 1 fb, peanuts
	6	BAR, active	None	None	WNL	49	3 chow, 2 apple, 8 veg, 4 df, 2 fb, peanuts
	7	BAR	None	None	WNL	48	4 chow, 1 apple, 3 veg, 7 df, 3 fb, peanuts
MMU-4 Female 24 Y.O	-1	BAR, timid	None	None	WNL	38	2 chow, ½ apple, 0 veg, 13 df, 2 fb, peanuts
	0	BAR	None	None	WNL	40	3 chow, 2 apple, 8 df, 5 fb, peanuts
	1	BAR	None	None	WNL, mild ketamine reaction	42	1 chow, 2 apple, 12 df, 4 df, 6 fb, peanuts
	2	BAR	None	Single dry cough	WNL, mild ketamine reaction	44	2 chow, 3 apple, 1 veg, 3 df, 2 fb, peanuts
	3	BAR	Clean nasal discharge w/ sneeze later	A couple of coughs at PM	Tachypnea without increased effort	48	3 chow, 3 apple, ½ banana, 3 fb, peanuts
	4	BAR, active	None at Obs, small amount later	Minor dried discharge on both nares	Mild nasal flaring	52	2 chow, 1 apple, 4 df, 2 fb, peanuts
	5	BAR	Mild dried discharges	None	Mild tachypnea	50	1 chow, 1 apple, ½ banana, 4 fb, peanuts
	6	BAR, pacing	None	None	Few episodes of quick shallow breath	44	2 chow, 1 apple, ½ banana, peanuts
	7	BAR	None	None	WNL	44	2 chow, 1 apple,2 df, 2 fb, peanuts
MMU-7 Female 22 Y.O	-1	BAR	None	None	WNL	39	2 chow, 2 apple, 1 veg, 9 df, 6 fb, peanuts
	0	BAR, active	None	Single dry cough	WNL	39	2 chow, 2 apple, 2 veg, 9 df, 2 fb, peanuts
	1	BAR, active	None initially, later clear	A couple of coughs AM	WNL	42	2 chow, 3 apple, 3 veg, 4 df, 4 fb, peanuts
	2	BAR	Small amount bilateral	Minor dry cough all day	Mild increase in abdominal component	48	4 chow, 1 apple, 2 veg, 5 df, 8 fb, peanuts
	3	BAR, active	None	Minor dry cough all day	Mild nasal flaring with each breath	52	3 chow, 1 apple, 2 veg, 5 fb, 3 fb, peanuts
	4	BAR	Mild dried discharges	Minor cough and sneezing all day	Mild nasal flaring with abd effort	54	2 chow, 2 apple, 1 veg, 4 df, 3 fb, peanuts
	5	BAR, active	None	Vet heard a single sneeze in the morning	Mild increased abd effort	50	4 chow, 2 apple, 1 veg, 7 df, 4 fb, peanuts
	6	BAR, pacing	None	Minor dry cough all day	WNL, quick shallow breath	48	3 chow, 1 apple, 1 veg, 6 df, 3 fb peanuts
	7	BAR	None	Isolated cough later in morning	WNL	44	2 chow, 2 apple, 1 banana, 4 df, peanuts
MMU-9 Female 18 Y.O	-1	BAR	None	None	WNL	41	4 chow, 2 apple, ½ banana, 3 fb, peanuts
	0	BAR, threats	None	None	WNL	37	2 chow, 1 apple, 12 df, 2 df, 2 fb, peanuts
	1	BAR	None	None	WNL, occasional quick shallow breath	39	2 chow, 1 apple, 6 veg, 5 df, 4 fb, peanuts
	2	BAR, threats	Mild dried discharge on nares	Dry cough during PM	Mild increase in abdominal effort	40	3 chow, 2 apple, 3 veg, 4 df, 9 fb, peanuts
	3	BAR	Mild discharges	A couple of coughs AM	Mild shallow breath	40	2 chow, 1 apple, 1/2 veg, 2 fb, 6 fb, peanuts
	4	BAR, anxious	Small amount bilateral	Minor dry cough all day	WNL, occasional quick shallow breath	39	4 chow, 1 apple, 1 veg, 5 df, 9 fb, peanuts
	5	BAR	Small amount bilateral	Minor dry cough all day	WNL, mild nasal flaring	41	3 chow, 2 apple, 4 veg, 8 df, 7 fb, peanuts
	6	BAR	Mild dried discharges	None	WNL, mild nasal flaring	40	4 chow, 1 apple, 3 veg, 5 df, 4 fb peanuts
	7	BAR, timid	Mild dried discharges	Single cough heard PM	WNL	39	3 chow, 2 apple, 1 banana, 4 df, peanuts
